# Physiological health Age (PhysAge): a novel multi-system molecular timepiece predicts health and mortality in older adults

**DOI:** 10.1007/s11357-025-01832-1

**Published:** 2025-09-01

**Authors:** Thalida Em Arpawong, Belinda Hernandez, Claire Potter, Robert J. Leigh, Eric T. Klopack, Claire Hill, Giovanni Fiorito, Laura J. Smyth, Aisling M. O’Halloran, Bernadette McGuinness, Jessica D. Faul, Rose Anne Kenny, Amy Jayne McKnight, Eileen M. Crimmins, Cathal McCrory

**Affiliations:** 1 Leonard Davis School of Gerontology, University of Southern California, Los Angeles, CA 90089 USA; 2 The Irish Longitudinal Study On Ageing (TILDA), Department of Medical Gerontology, School of Medicine, Trinity College Dublin, Dublin, Ireland; 3 Centre for Public Health, Queens University, Belfast, Northern Ireland; 4 Clinical Bioinformatics Unit, IRCCS Istituto Giannina Gaslini, Genoa, Italy; 5 School of Nursing and Midwifery, Trinity College Dublin, Dublin, Ireland; 6 Survey Research Center, Institute for Social Research, University of Michigan, Ann Arbor, MI 48106 USA

**Keywords:** Epigenetics, Accelerated aging, Mortality, Biological aging, Functional health

## Abstract

The complexity of epigenetic changes that accompany aging has been distilled into a number of molecular timepieces—termed epigenetic clocks—that characterize the pace of biological aging to differing degrees. Here, we develop and validate a DNA methylation-based Physiological health Age (PhysAge) score, comprised of eight DNA methylation surrogates to represent multi-system physiology and developed from commonly measured clinical biomarkers: CRP, peak flow, pulse pressure, HDL-cholesterol, Hba1c, waist-to-height ratio (WHR), cystatin C, and dehydroepianrosterone sulphate (DHEAS). We use data from the population-representative US Health and Retirement Study (HRS), split into a training (*n* = 1589) and test sample (*n* = 1588) and corroborate findings in two independent cohorts: The Irish Longitudinal Study of Aging (TILDA; *n* = 488) and the Northern Ireland Cohort for the Longitudinal Study of Ageing (NICOLA; *n* = 1830). PhysAge and the predominant second-generation epigenetic clocks, PhenoAge, GrimAge2, and DunedinPACE, were tested for their prediction of mortality and multiple age-related clinical measures (i.e., grip strength, gait speed, cognitive function, disability, frailty). PhysAge was comparable to extant clocks in predicting health measures and was indistinguishable from GrimAge2 in predicting mortality, despite not being trained on mortality. Moreover, the eight individual surrogates comprising PhysAge predicted health outcomes better than the measured values in many instances. The established clinical relevance of the biomarkers from which surrogates were derived opens up new opportunities for cross-study and cross-country comparisons of population health. Findings suggest that the DNA methylation PhysAge can be leveraged as a single biomarker to represent multiple physiological systems and offers utility in the context of clinical monitoring.

**Supplementary Information:**

The online version contains supplementary material available at 10.1007/s11357-025-01832-1.

## Introduction

The study of epigenetics is assuming increasing prominence in aging research globally [1–3]. The epigenome is a dynamic maintenance system that governs the transcription of the genome through a range of chemical modifications including histone modifications, non-coding ribonucleic acid (RNA), and deoxyribonucleic acid (DNA) methylation that regulate gene activity without altering the DNA sequence [4]. The epigenetic process most heavily researched in the context of aging is DNA methylation (DNAm), which refers to the addition or removal of methyl (-CH_3_) groups to cytosine-phospho-guanine (CpG) sites. There are ~ 28 million CpG sites in the human genome, and it is estimated that the methylation states of one-third of these sites change with age, becoming either hypo- or hyper-methylated [5]. Average levels of DNAm, across multiple cell types, at a relatively small number of CpG sites can be used to develop highly accurate measures of chronological age as well as age-related morbidity and mortality, termed epigenetic clocks. These molecular timepieces shaped by both genetic predispositions and environmental exposures can be used to track the passage of biological time, or accelerated passage of biological time, expressed as the difference between chronological age and DNAm age.

Horvath derived one of the first DNAm surrogate markers of chronological age with the development of the multi-tissue epigenetic clock based on levels of DNAm at 353 CpG sites [6]. This methodological approach has since been extended to include DNAm surrogates of blood-based clinical biomarkers that were trained on mortality such as PhenoAge or GrimAge [7, 8]. DNAm surrogates demonstrate immense promise for advancing precision medicine. One of the under-appreciated advantages of DNAm surrogates is that they allow for investigation of the relationship of an exposure with an outcome even if the specific exposure was not directly measured, but DNAm data are available. Indeed, developing DNAm surrogate markers is a growing enterprise and now includes surrogates for lead exposure[9], white blood cell proportions in blood [10], blood-measured proteins [11], and even electronic health-record-derived phenotypes such as medications, laboratory tests, and diagnoses [12].

Although it may seem counter-intuitive at first-blush, recent work suggests that DNAm surrogates may serve as stronger predictors of outcomes than actual measured levels of the biomarker [13], possibly because (i) they reflect individual differential responses to exposures and/or genetic susceptibility, (ii) they summarize the impact of multiple exposures (e.g., agents that cause inflammation) or multiple internal markers (e.g., inflammatory proteins), and (iii) the DNAm signature reflects the life history of the exposure more accurately than a single point in time measurement. For instance, Corley et al. [14] showed that a poly-epigenetic signature of smoking based on 230 CpG sites shows high correlations with phenotypic measures of smoking such as self-reported smoking status (*ɳ*^2^ = 0.63) and smoking pack years (*r* = 0.69), but predicts cognitive outcomes more strongly than the self-report measures. Of course, levels of DNAm at CpG sites across the methylome have the obvious advantage of not being subject to recall bias or dissimulation. More recently, investigators have shown that an algorithm derived from blood-based DNAm surrogate markers of cardiovascular disease (CVD) predicts short-term risk of cardiovascular events better than a prediction model based on traditional CVD risk factors [15].

Physiological health risk scores (PhysRS) are multi-system indices for quantifying the physiological “wear and tear” on the body that accrues over the life course due to a range of endogenous and exogenous exposures [16–19] and are associated with elevated risk for a range of adverse health outcomes and mortality. Recently, McCrory and collaborators [19] undertook an individual participant data (IPD) meta-analysis including data from 67,126 individuals aged 40–110 years in 13 cohort studies to develop a multi-system “state of physiological health” index. They examined the independent association of 40 different biomarkers across 12 physiological systems and identified a panel of eight biomarkers that were reliably and consistently associated with walking speed, grip strength, and self-reported health in the meta-analysis of these cohorts. The biomarkers included *C-reactive protein* (immune system), *heart rate* (cardiovascular), *high-density lipoprotein* (lipid), *waist-to-height ratio* (anthropometric), *glycated hemoglobin* (metabolic), *DHEAS* (neuroendocrine), *peak flow* (respiratory), and *cystatin C* (renal). The composite score derived from this brief panel of biomarkers was found to strongly predict mortality in each of the studies, and as such, may represent a measure of multi-system dysregulation for identifying biological age acceleration/biological vulnerability.

Developing DNAm surrogates of these established clinical biomarkers is of immense value to the field as it allows one to derive proxy measures of these exposures in cohorts in which they were not measured, opening up new opportunities for cross-study and cross-country comparisons of population health. Each clinical biomarker indexes a different aspect of physiological functioning, and, as such, can provide insight into which system may be contributing most to premature ageing, moving away from traditional approaches characterizing existing clock generation, which tend to be more agnostic regarding which biomarkers are retained in the predictive algorithm. Approaching clock development in this way also offers a way to monitor the efficacy of pharmacological, therapeutic, or lifestyle interventions at the individual level, as one can measure shifts in the individual biomarkers that contribute to meaningful changes in epigenetic age over time. In this paper, we describe the development and validation of a novel DNAm risk score—Physiological health Age (PhysAge) derived from DNAm surrogates of eight clinical biomarkers using data for 3177 participants in the US Health and Retirement Study Venous Blood Study (HRS-VBS). Illumina has recently introduced a new version of their methylation array (EPIC 2.0) and some of the sites constituting the established clocks have been lost in the transition to the successor chip: GrimAge will lose 183 of 1030, PACE will lose 29 of 173, and PhenoAge will lose 18 of 513 CpG sites. The impact of the omitted sites on the performance of the established clocks has yet to be established. We “future proof” our clock by only selecting sites common to EPIC 1.0 and EPIC 2.0. We examine the association of this physiological health state clock, PhysAge, with measured values of the biomarkers and other biological aging signatures, including DNAm-based measures of PhenoAge[20], GrimAge2[21], and DunedinPACE[22]. We compare its performance relative to the other clocks in predicting a broad range of age-related clinical phenotypes including grip strength, walking speed, self-rated health, frailty, disability, and mortality. We perform gene trait enrichment analysis to explore the functional significance of the genes that are regulated by the CpG sites comprising PhysAge to distil underlying biological pathways and their downstream effects. Finally, we validate the performance of the PhysAge clock in two independent nationally representative cohort studies on aging, the Irish Longitudinal Study on Ageing (TILDA) and the Northern Ireland COhort for the Longitudinal study of Ageing (NICOLA), to gauge the wider ecological validity of this new multi-system molecular timepiece.

## Results

**Fig. 1:**
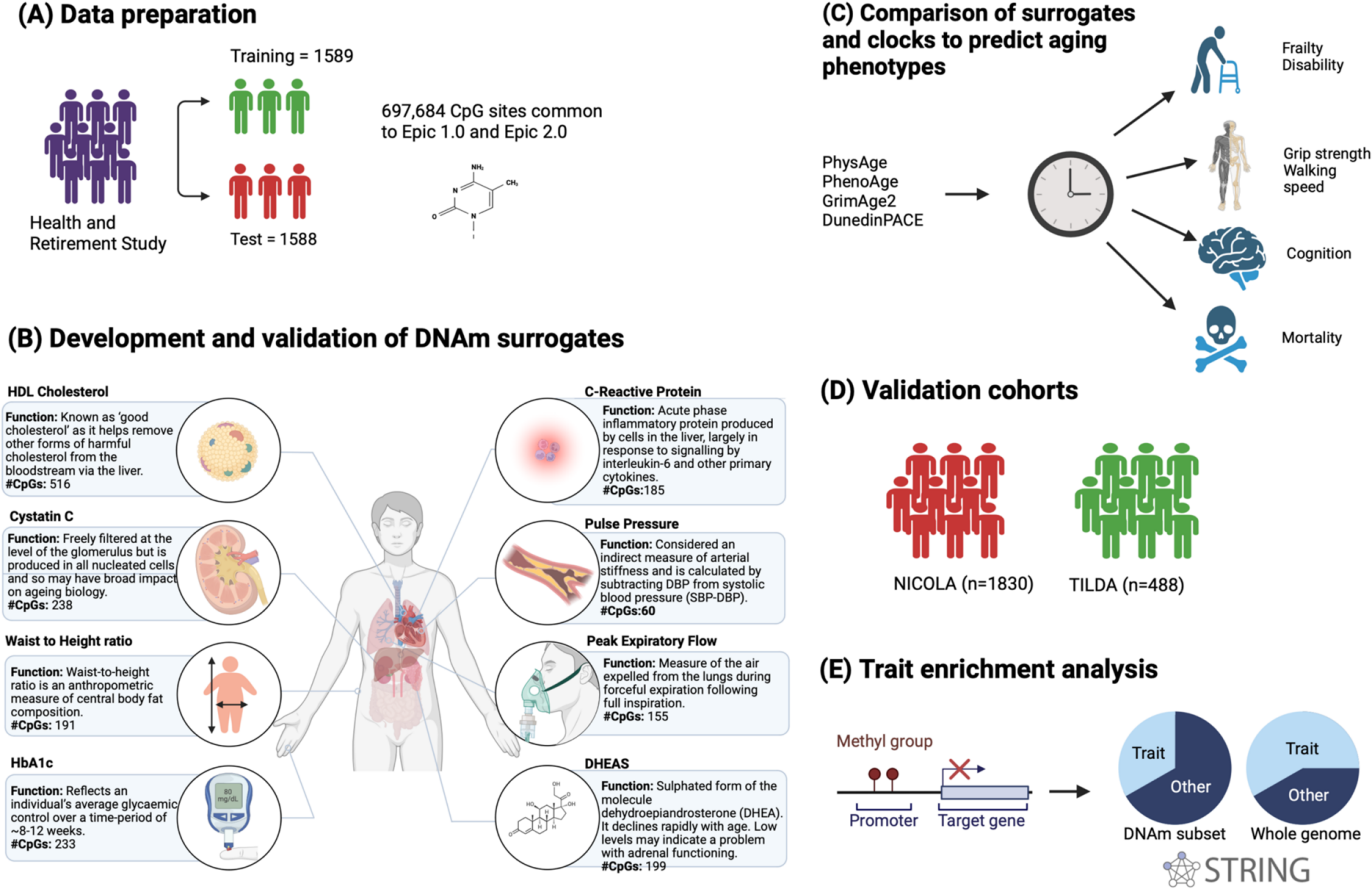
Workflow describing the development and validation of the DNA methylation physiological health age (PhysAge): (**A**) The Health and Retirement Study (HRS) methylation sample with complete data on biomarkers (*n* = 3177) was randomly split in half, *n* = 1589 training, and *n* = 1588 test sets. CpG sites not in common between the EPIC 1.0 and EPIC 2.0 chips were removed for greater translatability across other cohorts resulting in 697,684 CpG sites for analyses. (**B**) Elastic net regularization was used to develop an algorithm with DNAm levels predicting each of the eight biomarkers. CpGs identified and respective beta coefficients from the regularized prediction model were then used to calculate and validate the DNAm surrogate scores in the 50% test sample. Each DNAm surrogate was calculated as a sum of the weighted values for each CpG, then standardized (mean = 0, sd = 1). DNAm surrogates were then compared for their correlation with measured values. The PhysAge surrogate was then calculated as a sum of the eight DNAm surrogates, and the total score was standardized. DNAm PhysAge was then recalibrated to an age metric in years. (**C**) DNAm surrogates were evaluated for their prediction of age-related clinical phenotypes, and performance was compared to their measured biomarkers. The performance of PhysAge in predicting age-related clinical phenotypes was compared against PhenoAge, GrimAge2, and DunedinPACE. (**D**) The ability of DNAm PhysAge to predict ageing phenotypes was assessed in two independent validation cohorts—The Irish Longitudinal Study on Ageing (TILDA) and the Northern Ireland Cohort for the Longitudinal Study of Ageing (NICOLA).  (**E**) Gene enrichment analysis was undertaken with coding gene sets mapped from CpG sites comprising the DNAm surrogate biomarkers using StringDB *v*.12.0 and its associated databases. Created with BioRender.com

Figure 1 illustrates the methodological workflow involved in the development of PhysAge. For surrogate development, we randomly split the HRS-Venous Blood Sample that had complete data on all constituent biomarkers comprising PhysAge (*n* = 3177) into training (*n* = 1589) and test sets (*n* = 1588). The mean age of the training sample was 68.2 years (sd = 9.6), 57.5% were female, and from self-identification, were 67.0% Non-Hispanic White, 15.8% Non-Hispanic Black, 14.4% Hispanic/Latinx, and 2.8% Other. The mean age of the test sample was 68.2 years (sd = 9.8), 58.5% were female (Table 1), and 68.1% Non-Hispanic White, 15.2% Non-Hispanic Black, 14.2% Hispanic/Latinx, and 2.5% Other.

**Table 1:** Summary statistics for the biological aging measures and health outcomes in the HRS test sample (*n* = 1588)

	Mean	SD	Min	Max	Description
Biological aging measures					
Age	68.16	9.75	49.0	93.0	Chronological age
Female sex	58.5%	**-**	**-**	**-**	
PhysRS	0.00	3.82	− 11.92	18.60	Sum of the eight standardized measured variables
PhysAge surrogate	0.00	4.45	− 13.33	18.31	DNAm surrogate sum of the standardized eight-item PhysAge
PhysAge	68.15	9.63	39.32	107.75	DNAm PhysAge
PhenoAge	57.48	10.08	26.72	101.68	DNAm PhenoAge
GrimAge2	72.70	8.97	49.39	105.25	DNAm GrimAge
DunedinPACE	1.03	0.15	0.69	1.74	DNAm DunedinPACE
zPhysRS_AA	0.00	1.00	− 3.12	4.87	Standardized age-residualized version of the measured Physiological health Risk Score
zPhysAge_AA	0.00	1.00	− 3.22	4.60	Standardized age-residualized version of the DNAm surrogate
zPhenoAge_AA	0.00	1.00	− 3.54	6.00	Standardized age-residualized version of PhenoAge
zGrimAge2_AA	0.00	1.00	− 2.89	5.07	Standardized age-residualized version of GrimAge2
zDunedinPACE	0.00	1.00	− 2.10	3.73	Standardized age-residualized version of DunedinPACE
Health outcomes					
Walking speed (cm/s)	79.90	23.90	8.07	125.10	Average of two-timed walks over a distance of 98.5 inches
Mean grip strength (kg)	30.57	10.82	2.00	100.00	Highest recorded grip strength of four readings
Self-Rated Health = fair/poor	27.1%	-	-	-	Excellent/V good/good = 0; fair/poor = 1
ADLs	0.00	0.76	0.00	5.00	ADLs included difficulties with (1) dressing; (2) walking across a room; (3) bathing or showering; (4) eating, such as cutting up food; (5) getting in or out of bed
IADLs	0.00	0.69	0.00	5.00	IADLs included (1) difficulties in preparing a hot meal; (2) shopping for groceries; (3) making telephone calls; (4) taking medications, and (5) managing money
Frailty	21.31	14.10	0.64	79.62	39-item frailty index
TICS errors	11.92	4.30	0.00	27.00	TICS cognitive score, as the number correct subtracted from the total possible score of 27
Mortality	18.1%	-	-	-	Hazard of all-cause mortality from 2016 to 2022 follow-up

### Development of the DNAm surrogates and Physiological health Age (PhysAge) score

A two-stage process was employed to develop and validate DNAm surrogates for the eight biomarkers that were selected for their utility as screening tools to identify pre-clinical health states for each physiological system (McCrory et al., 2023). In step 1, the training sample was used to determine tuning parameters of alpha and lambda for each measured biomarker. In step 2, elastic net regularization was used to develop an algorithm with DNAm levels predicting each biomarker. The regularized prediction models identified relevant CpGs and respective beta coefficients used to calculate the DNAm surrogates with 60 CpGs for pulse pressure, 155 for peak flow, 191 for WHR, 233 for HbA1c, 185 for CRP, 238 for cystatin C, 199 for DHEAS, and 516 for HDL ratio (Fig. 1).

In the test sample, for each biomarker, the sum of the weighted values for identified CpGs were used to calculate the respective DNAm surrogate. Each DNAm surrogate was standardized; then, all eight surrogates were summed to produce the DNAm surrogate for PhysAge. We then transformed the standardized DNAm surrogate for PhysAge into a parameter to reflect age in years by using the mean and standard deviation of age in the training set. This yielded a DNAm PhysAge score of 68.2 (sd = 9.6) with a range of 39.3 to 107.8 years in the test set. We tested the correlations between the measured biomarkers with their equivalent DNAm-based surrogate biomarker in the HRS test data (SI Appendix Figure S1a). The average correlation was 0.44, ranging from a low of 0.30 for DHEAS to a high of 0.64 for peakflow. Because the surrogate for heart rate correlated poorly with measured values (*r* = 0.07), we developed an alternative measure of cardiovascular system dysregulation by calculating a DNAm surrogate of pulse pressure (systolic blood pressure–diastolic blood pressure), which correlated 0.32 with measured values. We observed that each DNAm surrogate showed overall specificity in predicting its clinical biomarker vs other biomarkers (SI Appendix 1, Figure S10). For six out of eight surrogates (HbA1c, CRP, cystatin C, HDL, WHR, and peakflow), closer ties are observed for each surrogate with its target biomarker vs other biomarkers (*r* 0.29 to 0.64). The two exceptions are the surrogate for DHEAS, which is correlated with its clinical measure (*r* = 0.30) and also with peakflow (*r* = 0.39), and the surrogate for pulse pressure, which is correlated with its clinical measure (*r* = 0.32) and also with cystatin C (*r* = 0.39). Additionally, a strong pattern of correlations emerged between the surrogates and the specific health parameters they are intended to predict, for example, DNAm HbA1c and ever having been diagnosed with type 2 diabetes (*r* = 0.28), DNAm WHR and high BMI (*r* = 0.39), DNAm CRP and inflammatory markers (*r* = 0.36 with Interleukin-1 receptor antagonist (IL1RA), *r* = 0.33 with interleukin-6 (IL6)), or DNAm HDL and total cholesterol (*r* = 0.30; SI Appendix 1, Figure S11).

**Fig. 2:**
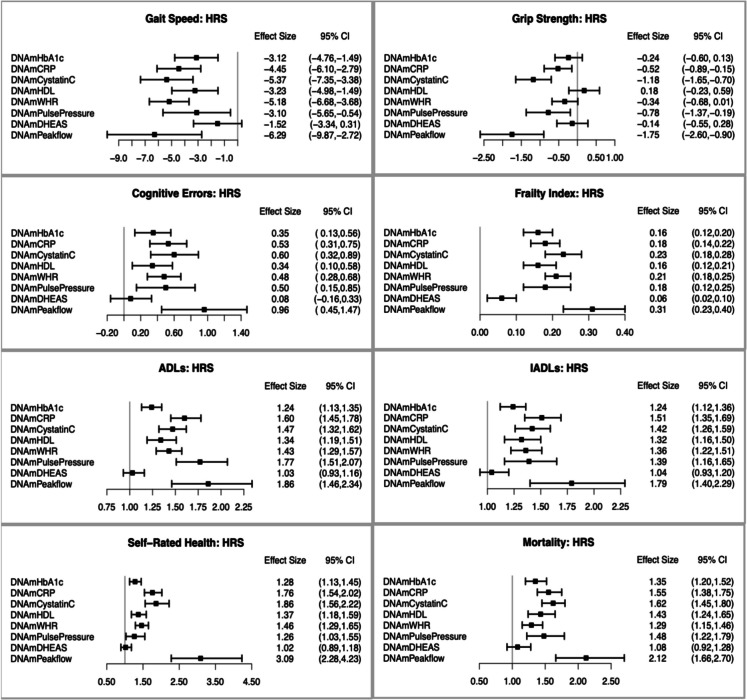
Associations of the individual DNA methylation surrogate biomarkers comprising the DNA methylation Physiological health Age score (PhysAge) with a range of clinical phenotypes in the HRS test data. Effect sizes are displayed as beta values for walking speed, grip strength, cognitive errors, and frailty; incident rate ratios for ADLs, IADLs, and Self-Rated Health; and Hazard Ratios for Mortality. Scales for DNAm surrogates of HDL, DHEAS, and peakflow are reversed so that all surrogates are expected to show the same direction of effect with the phenotypes. All associations are adjusted for age, sex, ethnicity, and white blood cell counts. Associations with gait speed and grip strength were also adjusted for height

**Fig. 3:**
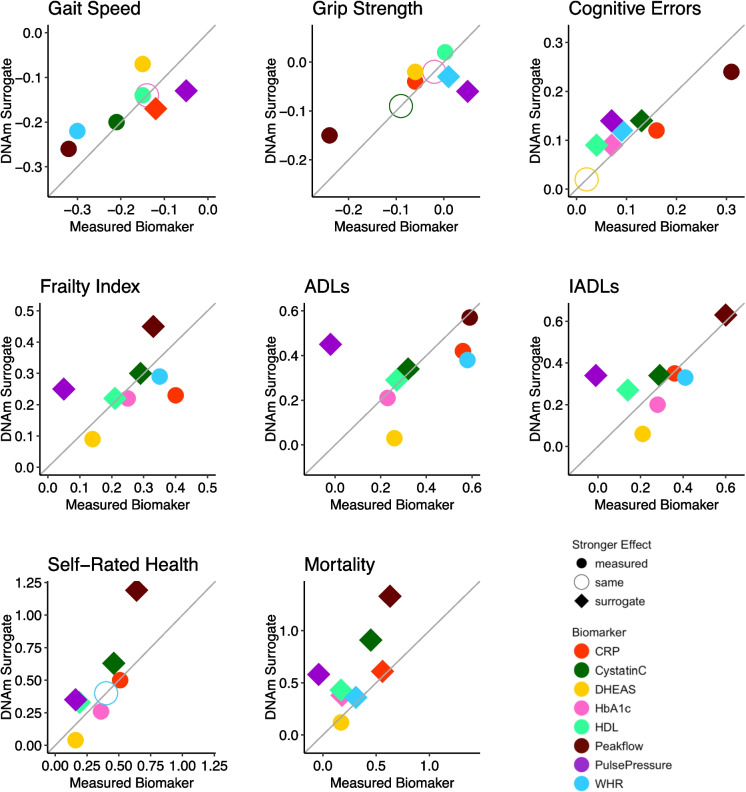
Comparing prediction of aging phenotypes from eight DNAm surrogates and measured biomarkers in HRS. Each of the eight panels focuses on one of the aging phenotypes and shows standardized effect sizes for phenotype prediction from both the measured biomarker (*x*-axis) and the biomarker DNAm surrogate (*y*-axis). The gray diagonal line shows where *X* = *Y*, and thus, prediction from measured and DNAm biomarker would be equivalent. Biomarkers are indicated by a specific colour and a filled diamond where prediction from the surrogate is stronger than the measured biomarker in the expected direction of effect, or a filled circle where the measured biomarker is stronger and a hollow circle where the effects of both are the same. Scales for HDL, DHEAS, and peakflow are reversed so that all surrogates and biomarkers are expected to show the same direction of effect with the phenotypes. All models were run with each DNAm surrogate or biomarker separately on the outcome, adjusted for age, sex, and race/ethnicity. Models for gait speed and grip strength were also adjusted for height

The association of each of the eight individual surrogates comprising PhysAge with the aging phenotypes in HRS is shown in Fig. 2 (TILDA and NICOLA are shown in SI Appendix 1, Figure S4a and S4b). Peakflow was very strongly associated with all aging phenotypes, as was CRP, cystatin C, and pulse pressure. HDL, WHR, and HbA1c were associated with seven outcomes (all except grip strength), while DHEAS was the worst performing and predictive only of frailty. We also compared the relative performance of the eight measured biomarkers and their DNAm surrogates in predicting aging phenotypes, as shown in Fig. 3 for the HRS (TILDA and NICOLA comparisons shown in SI Appendix 1, Figure S2a and S2b). Figure 3 shows that in the HRS test set, DNAm surrogates predicted more strongly than the measured biomarkers for all phenotypes in 31/64 comparisons and perform as well for another 5/64 comparisons. Remarkably, all DNAm surrogates predicted mortality more strongly compared to the measured biomarkers, except for DHEAS, for which prediction was nearly equivalent. Overall, DNAm surrogates and measured biomarkers showed highly similar effects for HbA1c, CRP, cystatin C, HDL, WHR, and peakflow across aging phenotypes. There were two exceptions with the DNAm surrogate for pulse pressure performing more strongly for all phenotypes, and conversely, the measured biomarker for DHEAS performing more strongly for all phenotypes except for cognitive errors, for which performance was equivalent.

PhysAge shares three surrogates in common with the GrimAge2 clock although they were developed by different investigators and using different cohorts. DNAm surrogates in common between PhysAge and GrimAge2 correlate highly (DNAmCRP *r* = 0.57, DNAmCystatinC *r* = 0.70, and DNAmHbA1c *r* = 0.55), despite there being very few CpG sites in common between the surrogates (DNAmCRP has 3, DNAmCystatinC has 2, and DNAmHbA1c has 4 (SI Appendix 1, Table S3)). Comparing the prediction of aging phenotypes from surrogates from both PhysAge and GrimAge, they show nearly identical prediction across outcomes (SI Appendix 1, Figure S6) despite the differences in construction.

### Comparison of clocks to predict mortality and health outcomes

**Fig. 4:**
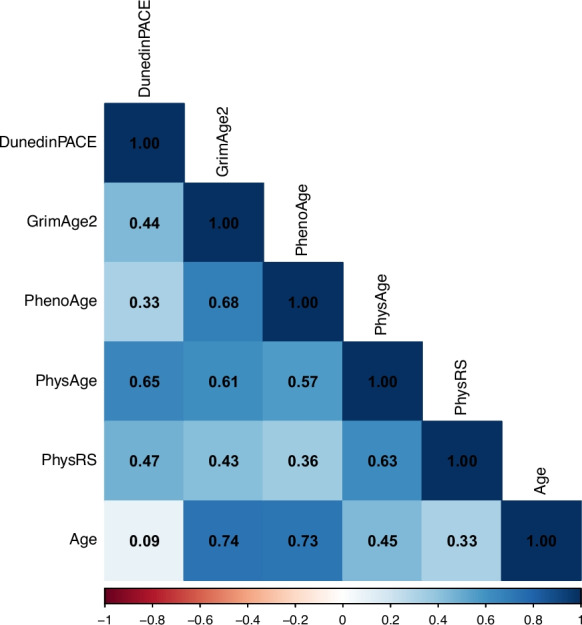
Pearson intercorrelations of the different biological aging measures in the HRS test set

The next step in the analysis was to consider the relation of DNAm PhysAge with measured values, chronological age, and the extant second-generation epigenetic clocks. We used the measured biomarkers to calculate a Physiological health Risk Score (PhysRS) by standardizing scores on each of the eight measured biomarkers, then summing the eight standardized values. As there were several biomarkers (DHEAS, peakflow, and HDL) for which higher values indicate lower risk, we reversed the scores by multiplying by − 1 prior to standardizing. DNAm PhysAge correlated 0.63 with the measured PhysRS (Fig. 4) and 0.45 with chronological age. The correlations of DNAm PhysAge with the second-generation clocks were in the moderate range and slightly higher for DunedinPACE (*r* = 0.65) compared with GrimAge2 (*r* = 0.61) and PhenoAge (*r* = 0.57).

**Fig. 5:**
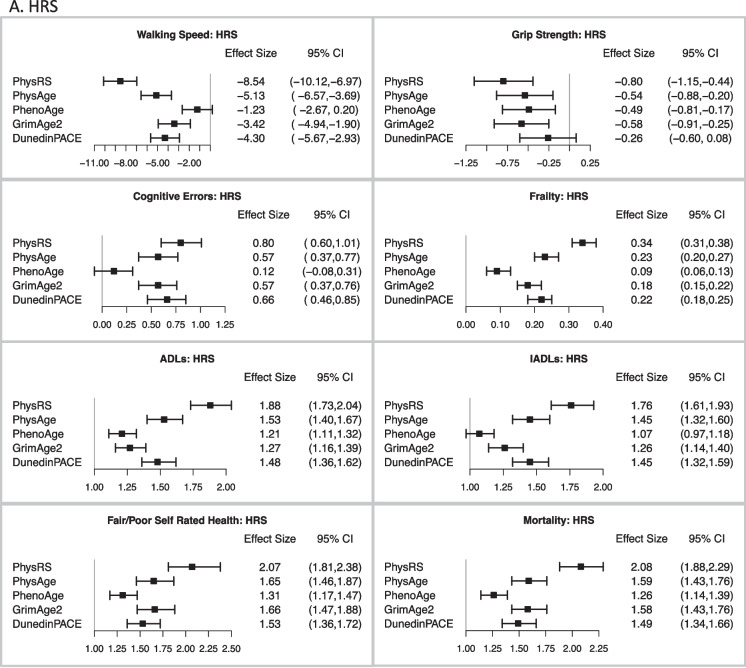

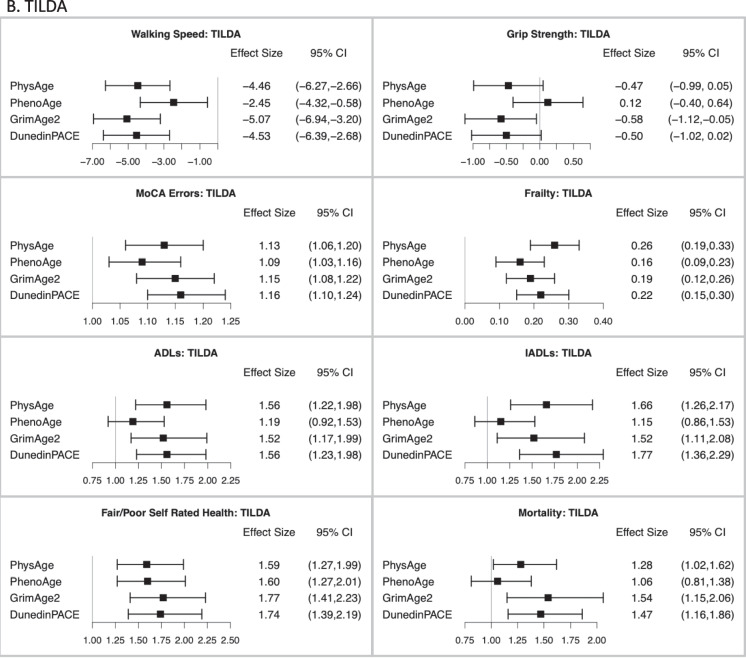

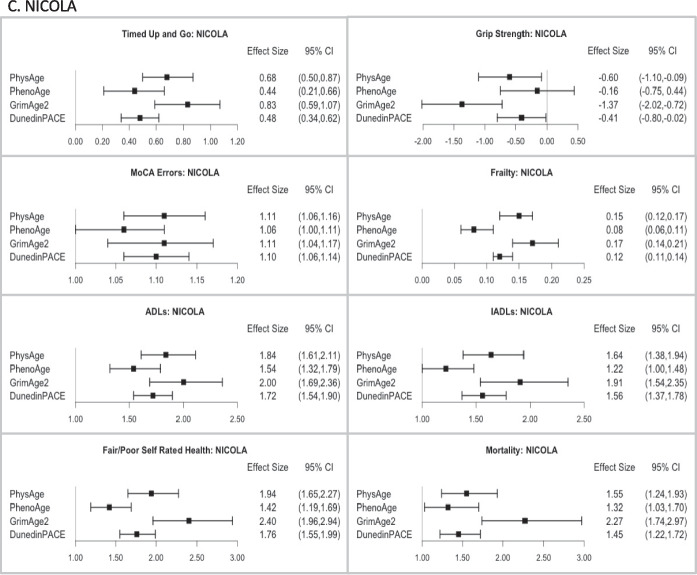
Association of the measured and different methylation risk scores with a range of clinical phenotypes in the **A** HRS test data, *n* = 1588; **B** TILDA, *n* = 488; and **C** NICOLA, *n* = 1830. Effect sizes are displayed as beta values for gait speed, grip strength, cognitive errors, and frailty; incident rate ratios for ADLs, IADLs, and Self-Rated Health; and Hazard Ratios for Mortality. All associations are adjusted for age, sex, ethnicity, and white blood cell counts. Associations with walking speed and grip strength were also adjusted for height

Figure 5 illustrates the comparative performance of the different epigenetic measures and measured PhysRS in predicting a host of age-related clinical phenotypes, adjusting for age, sex, ethnicity, and white blood cell counts. Figure 5 a shows that in the HRS test sample, PhysAge is a strong predictor of all age-related clinical phenotypes. A standard deviation increase in PhysAge was associated with significantly lower grip strength [*b* =  − 0.54; − 0.88, − 0.20] slower walking speed [*b* =  − 5.13; − 6.57, − 3.69], frailty [*b* = 0.23; 0.20, 0.27], and with measures of disability, including limitations in activities of daily living (ADLs) [IRR = 1.53; 1.40, 1.67] and instrumental activities of daily living (IADLs) [IRR = 1.45; 1.32, 1.60]. It was also associated with worse self-rated health [OR = 1.65; 1.46, 1.87], errors on a global cognitive battery [b = 0.57; 0.37, 0.77], and all-cause mortality over a mean 5.3-year follow-up [HR = 1.59; 1.43, 1.76]. Notably, PhysAge predicted all-cause mortality at the same level as GrimAge2 [HR = 1.58; 1.43, 1.76], and better than both PhenoAge [HR = 1.26; 1.14, 1.39] and DunedinPACE [HR = 1.49; 1.34, 1.66].

We used the area under the curve (AUC) to assess whether PhysAge represents an improvement in predictive performance relative to the PhysRS and other extant second-generation clocks in identifying age-related decline or mortality. Using the median value to dichotomize continuous variables, we interpret the AUC as the probability that a predictor would correctly classify an individual into the correct group. All else being equal, a higher AUC indicates stronger prediction, with values of 0.7–0.8 considered acceptable and 0.8 or higher considered excellent. This analysis in the HRS test data revealed that although PhysRS was slightly stronger at prediction overall with AUCs between 70.8 to 89.7, PhysAge AUCs ranged from 67.8 to 89.5 across phenotypes. Other clocks yielded very similar AUCs to PhysAge (GrimAge2 66.3 to 89.5, PhenoAge 62.8 to 89.6, and DunedinPACE 66.3 to 89.5) with no clocks differing more than 5% in prediction of the same phenotype (SI Appendix 1, Table S2 and Figure S5). The majority of differences in AUC between PhysAge were with PhenoAge (2–5%), whereas nearly all differences with GrimAge2 or DunedinPACE were < 2%, including when predicting mortality. All clocks were nearly indistinguishable when predicting grip strength and cognitive errors.

### Validation cohorts

The next step was to determine how well the new PhysAge clock performed in two independent validation samples comprising 488 individuals participating in TILDA, and 1830 individuals in NICOLA, for whom DNAm data was available. Table S1b and S1c report the baseline characteristics for the TILDA and NICOLA epigenetic samples, respectively. The mean age of the TILDA and NICOLA epigenetic samples was 62.2 (SD = 8.35) and 64.5 (SD = 9.09), respectively, with equal representation of males and females. TILDA and NICOLA measured six of the eight biomarkers [all except DHEAS and peak flow] that were used to develop PhysAge in the HRS-VBS. The correlation of the measured biomarkers with the DNAm surrogate markers in TILDA and NICOLA are illustrated in Figures S1b and S1c, respectively (SI Appendix 1, Figure S1). In TILDA, the average correlation was 0.38, ranging from a low of 0.28 for HbA1c to a high of 0.48 with cystatin-C. PhysAge correlated 0.53 with chronological age, while the correlation with the clocks was PhenoAge (*r* = 0.65), GrimAge2 (*r* = 0.72), and DunedinPACE (*r* = 0.69) (SI Appendix 1, Figure S3a). In NICOLA, the average correlation of the measured values with the DNAm surrogates was remarkably similar at 0.35; lowest, once again, for HbA1c (*r* = 0.26) and highest with HDL and cystatin C (*r* = 0.47). PhysAge correlated 0.49 with chronological age in NICOLA, while the correlation with the clocks was PhenoAge (*r* = 0.77), GrimAge2 (*r* = 0.60), and DunedinPACE (*r* = 0.71) (SI Appendix 1, Figure S3b).

The same eight outcome variables used to validate DNAm surrogates and PhysAge in the HRS test data were also available in TILDA and NICOLA [described in SI Appendix 1], although performance on the timed-up-and-go (TUG) task was used as a substitute for walking speed in NICOLA. When comparing the relative performance of the DNAm surrogates and their measured biomarkers in the TILDA and NICOLA cohorts (SI Appendix 1, Figure S2a and S2b), we observe that, for the most part, they perform similarly in predicting all outcomes. In TILDA, the surrogates predict better than measured biomarkers for 29/48 comparisons, and predicted equivalently for another three comparisons. In NICOLA, the surrogates predict better than measured biomarkers for 34/48 comparisons. Particularly with respect to mortality, all DNAm surrogates performed better than the measured biomarkers except for cystatin C and HDL in TILDA and except for the HbA1c and HDL surrogate in NICOLA. Additionally, supplementary figures S4a and S4b (SI Appendix 1, Figure S4) show the association of the individual surrogates with the aging phenotypes in TILDA and NICOLA respectively. We observe a pattern whereby the DNAm surrogates show very similar patterns of effects to HRS, suggesting that the individual DNAm surrogates have broad ecological validity when applied in other aging cohorts.

Next, as shown in Fig. 5 b and c, PhysAge strongly predicted the aging phenotypes in both cohorts. A standard deviation increase in PhysAge was associated with lower grip strength [TILDA: *b* =  − 0.47; − 0.99, 0.05] [NICOLA: *b* =  − 0.60; − 1.10, − 0.09]; slower walking speed [TILDA: *b* =  − 4.46; − 6.27, − 2.66] and slower TUG time [NICOLA: *b* = 0.68; 0.50, 0.87]; frailty [TILDA: *b* = 0.26; 0.19, 0.33] [NICOLA: *b* = 0.15; 0.12, 0.17]; and with measures of disability, including ADL’s [TILDA: IRR = 1.56; 1.22, 1.98] [NICOLA: IRR = 1.84; 1.61, 2.11] and IADLs [TILDA: IRR = 1.66; 1.26, 2.17] [NICOLA: IRR = 1.64; 1.38, 1.94]. PhysAge was also associated with worse self-reported health [TILDA: OR = 1.59; 1.27, 1.99] [NICOLA: OR = 1.94; 1.65, 2.27], errors on a global cognitive battery [TILDA: OR = 1.13; 1.06, 1.20] [NICOLA: OR = 1.11; 1.06, 1.16], and with all-cause mortality [TILDA: HR = 1.28; 1.02, 1.62] [NICOLA: HR = 1.55, 1.24, 1.93]. The performance advantage or equivalence of PhysAge over PhenoAge seen in the HRS test data, as assessed using AUC probabilities (SI Appendix 1, Table S2a and Fig. 5a), was also observed in TILDA and NICOLA (SI Appendix 1, Table S2b–S2b and Fig. 5b–c). In both TILDA and NICOLA, PhysAge was nearly interchangeable with GrimAge2 and DunedinPACE in the AUC prediction for all outcomes, including mortality.

### Gene trait enrichment analysis

CpG sites in PhysAge were mapped to genes using the Illumina manifest for the EPIC 1.0 array. Then, we conducted gene trait enrichment analysis using StringDB *v*.12.0 to determine whether gene sets identified by the CpG sites implicated functional properties at higher proportions than expected by chance. When all CpG sites for the eight DNAm surrogates were considered together, a large number of corresponding gene-trait enrichments were observed. In broad terms, enrichments were found in regulatory elements (with 776 of 1039 genes) and for anatomical development (with 361 of 1039 genes). Furthermore, genes involved in five cancer specific pathways were enriched. Interestingly, even more whole-body expression (830 of 1039), phosphoprotein (591 of 1039), and alternative splicing genes (713 of 1039) were observed (SI Appendix 1, Figure S7 & Dataset S1).

## Discussion

### A new DNA methylation molecular timepiece

We sought to develop a DNA methylation-based physiological health age (PhysAge) for quantifying multi-system physiological dysregulation. We first developed DNAm surrogates for a panel of eight clinical biomarkers predictive of functional health using data from the HRS. In the second step, we standardized scores for each of the surrogates and summed them to derive the PhysAge clock. The correlation between the measured physiological health risk score (PhysAgeRS) and DNAm PhysAge was substantial at 0.63 and attests to the viability of using DNAm surrogates in instances where an exposure has not been measured. This conclusion is further buttressed by the finding that PhysAge predicts a broad range of health outcomes in the HRS test data and in two independent validation cohorts (TILDA and NICOLA) and does so as well as the current “best in class” second-generation clocks including GrimAge2 and DunedinPACE. One notable advantage of PhysAge over the other clocks is that it was “future proofed” by selecting on sites common to EPIC 1.0 and EPIC 2.0, whereas the previously developed clocks will be affected to a greater or lesser degree with the transition to EPIC 2.0 [23, 24] and will likely require some level of statistical re-weighting or imputation for missing sites. Additionally, PhysAge was trained on a nationally representative population with socioeconomic and ancestral diversity, which increases its portability across cohorts and countries.

We also observed substantial correlations (*r* = 0.6–0.7) of PhysAge with the other second-generation epigenetic clock estimators even though they were built in different ways and using different compositions of biomarkers. Levine’s innovation in producing the first of the second-generation clocks was to include clinical biomarkers when developing her phenotypic age estimator and to seek the DNAm signature for that composite measure. Lu adopted a similar approach but focused on inflammatory proteins in addition to smoking pack years when calibrating the GrimAge2 clock. The DunedinPACE clock was developed by identifying DNAm correlates at a single time point of decline in 19 indicators of organ-system integrity across four measurement occasions in a cohort of same aged individuals. In this paper, we developed a DNAm measure calibrated from eight DNAm surrogates of established clinical biomarkers. This would seem to imply that the reason why the second-generation epigenetic clocks perform so well is that DNAm is clearly very adept at picking up the age-related physiological deterioration that occurs across multiple bodily systems in aging.

### Overlap and distinctness in the design of the second generation epigenetic clocks

There is a low degree of overlap between the CpG sites comprising PhysAge and the other extant second-generation clocks (SI Appendix 1, Figure S9). In total, there are 1711 CpG sites in PhysAge with 66 of them involved in two or more surrogates. PhysAge shares 10 sites in common with PhenoAge, 33 in common with GrimAge, and 12 in common with DunedinPACE. No CpG sites were common to all four clocks, although five CpG sites were shared between PhysAge, GrimAge2, and DunedinPACE. If we compare PhysAge, GrimAge2, and DunedinPACE, we see some direct and indirect similarities in the composition of the clinical biomarkers used in their construction. PhysAge overlaps entirely with GrimAge2 for three biomarkers—CRP, glycated hemoglobin (HbA1c), and cystatin C. They both also include a measure of HPA-axis functioning (DHEAS vs adrenomedullin), lipid functioning (HDL vs leptin), and cardio-respiratory fitness (peakflow vs smoking pack years). Similarly, PhysAge overlaps entirely with DunedinPACE for three biomarkers—CRP, HbA1c, and HDL—and has close analogues for measures of anthropometric fitness (waist-to height ratio vs weight-hip ratio), cardio-respiratory fitness (peak flow vs forced vital capacity ratio), blood pressure (pulse pressure vs systolic blood pressure), and renal functioning (cystatin C vs estimated glomerular filtration rate). It is perhaps not surprising therefore that these clocks seem to be more or less interchangeable in their ability to predict a broad range of age-related clinical phenotypes measured in these cohorts.

PhenoAge, by contrast, was less strongly related to the health outcomes. Consideration of what it lacks relative to the other clocks may provide further insights into the biology of the aging process. While PhenoAge included CRP, glucose, and creatinine in its development, it does not include a measure of lipid metabolism nor of cardio-respiratory fitness, which are prominent features of the other clocks. Moreover, creatinine has been shown to be less sensitive than cystatin C in predicting functional loss [25] and in estimating the glomerular filtration rate in older age samples [26]. Additionally, GrimAge2 and PhysAge incorporate cardio-respiratory metrics, with smoking pack-years in GrimAge2 and peakflow in PhysAge, both of which have strong associations with clinical phenotypes. However, the broader utility of PhysAge lies in the design and selection of biomarkers, included for their specific ability to predict pre-clinical health states for targeted physiological systems.

A recent paper demonstrated how patterns of responsiveness to different pharmacological and lifestyle interventions could be indexed by specific DNAm clocks [27]. Clocks that were derived from sub-scores were called “explainable clocks” and provided more valuable mechanistic insights to the responsiveness patterns compared to single aggregate clocks. In parallel, PhysAge with its component surrogates provides more direct interpretability as a marker for tracking physiological health state related to system functioning. A clock constructed in this manner from biomarkers with established clinical cut-points has utility in the context of clinical trial monitoring. For instance, if PhysAge shifts in response to a pharmaceutical intervention, we can decompose the clock to look at the constituent systems (immune, cardiovascular, lipid, anthropometric, metabolic, neuroendocrine, respiratory, renal) using its DNAm surrogates to see which biomarkers are contributing most to the changes in the “ticking rate” of the PhysAge clock, moving towards precision health. An example of this type of profiling is demonstrated with simulated data, generated to reflect properties of the HRS test set. Simulated data for DNAm PhysAge and its constituent DNAm surrogates show that when they are expressed in the age metric of years for a random sample of four men (M1-M4), who have the same estimated DNAm PhysAge of 60 years, drivers of accelerated PhysAge can be identified (SI Appendix 1, Table S5). For instance, M1 has a calendar age of 77.4 but an estimated PhysAge of 60.6. The only biomarker on which they show age acceleration is pulse pressure. M4 by comparison has a calendar age of 52.8 and a PhysAge of 60.8. They display age deceleration for one biomarker (peakflow) and age acceleration for seven biomarkers. This may point towards facets of their health that warrant closer investigation based on the methylation readout. We show a similar example for four women (W1–W4), who have the same estimated PhysAge of 80. W3 has a calendar age of 71.5 and a PhysAge of 80.8 and is characterized by quite dramatic age acceleration across five surrogates (HbA1c, CRP, DHEAS, pulse pressure, peakflow) and age deceleration across three surrogates (cystatin C, HDL, and WHR). These surrogate ages might identify this individual as “at risk” of poor outcomes and suggests further investigation may be warranted as well as identifying potential areas for improvement.

### Signaling information loss may be a feature of aging

Gene-trait enrichment analysis is a bioinformatic technique used to determine whether a given subset of genes are enriched for a biological function compared with all other genes in the organism. Analysis of the DNAm surrogates revealed the presence of enriched whole bodily expressed phosphoproteins and alternative splicing genes, suggesting that the combined surrogate set consists of diverse, regulatory, and multifunctional proteins involved in a multitude of pathways, specifically involved in signaling and development (SI Appendix 1, Figure S7). These results suggest that our DNAm surrogate sets correlate strongly with signalosomal desynchronization (disruption of cell–cell communication, considered a hallmark of aging [28]) and the impaired activity of pathways.

### Epigenetic clocks as molecular crystal balls

The epigenetic clocks have gained enormous traction amongst the Geroscience community in recent years because DNAm is highly stable and yields consistently stronger correlations with chronological age than alternative single omics-based platforms such as metabolomics, transcriptomics, or proteomics[29]. Moreover, the metric of the epigenetic clock—expressed as years of age acceleration/deceleration is at once appealing and intuitive because it is essentially the difference between the estimated DNAmAge and the number of years one has lived to date. Nevertheless, the fact that the sum score of the measured values predicted the majority of outcomes as well or more strongly than the epigenetic clocks (Fig. 5) indicates that DNAm remains just one amongst a host of potential indicators of biological aging. One might even conclude that the second-generation clocks are just a proxy for the physiological dysregulation that is already captured by clinical biomarkers at a fraction of the cost of running DNAm analysis and associated bioinformatic pipelines. While it is abundantly clear that changes in DNAm are strongly associated with chronological aging, it remains to be determined whether they cause aging or are simply a bioproduct of the aging process[30], akin to greying hair, skin wrinkling, or gum erosion.

Given these caveats, what then is the value of PhysAge and our approach to clock generation? In short, DNA surrogate markers have the potential to revolutionize the field of population health as we can derive proxies for these exposures in studies where they were not measured, but where methylation data is available. The results of this study attest to the efficacy of the enterprise as we have shown that the surrogates predict health outcomes better in many instances than the measured values of the biomarker, likely because they better reflect life histories of exposure and accumulated physiological dysregulation. We also anticipate applications for these surrogates in cohorts in which the clinical biomarkers would not be routinely measured (e.g., younger cohorts), thereby allowing us to track how the ageing process unfolds over time and at earlier life stages. For example, HDL-cholesterol and glycated hemoglobin are not routinely measured in healthy young adults, but we would now have surrogates for them.

While the current belief is that epigenetic changes reflect rather than precede changes in biological age, longitudinal data are urgently required to disambiguate a relationship that is necessarily confounded in cross-section, particularly given the growing movement towards using epigenetic clocks as surrogate endpoints in clinical trials, sometimes in the absence of any great understanding of what it is that the clocks are measuring [31]. For instance, some small-scale recent studies in humans suggest that epigenetic aging is reversible [32, 33]. This is perhaps unsurprising as the interventions they target (e.g., dietary changes) will usually lead to changes in the levels of many of the biomarkers (e.g., HDL, leptin, triglycerides, inflammation) that were used in the construction of the second-generation clocks, but it is not yet clear whether this leads to any notable change in the risk of developing non-communicable diseases or morbidity [34]. In short, age reversion may be more apparent than real. Moreover, the magnitude of the baseline and post-intervention change in epigenetic aging in these trials can sometimes seem very large for a short-term [8-week] intervention relative to the long-run effects of something like life course socio-economic hardship [35–39], maltreatment or abuse [40], or even to measurement error from the epigenetic arrays. Finally, it should be acknowledged that epigenetic changes constitute only one of the three mooted primary hallmarks of aging, which also includes genomic instability and telomere erosion [28]. Further work is needed to unravel the inter-relation of these age-related alterations in function to develop the sort of integrative multi-omics conceptual model that Geroscience has been searching for but has not yet managed to unearth[29, 41].

## Conclusions

We have shown that it is possible to derive a DNAm-based physiological health age indicator that is indicative of functional loss, frailty, disability, and mortality using DNAm surrogate markers of established clinical biomarkers. The epigenetic clocks seem to provide a useful tool for profiling differences in the pace of biological aging between same-aged individuals, and as such, may have utility as surrogate endpoints in clinical trials where they can be used to assess the efficacy of lifestyle, pharmacologic or other therapeutic anti-aging interventions(42, 43). The epigenetic clocks have performed an important function by distilling a hugely complex system—approximately 28 million CpG sites in the human methylome—into a remarkably accurate biomarker of aging, but the next frontier is to move beyond single point-in-time measures to more fully elucidate how temporal shifts in DNAm at particular CpG sites contribute to individual differences in health span and life span. Interestingly, a recent paper which used mendelian randomization to identify CpG sites implicated in the aging process concluded that none of the existing clocks showed significant enrichment for causal CpGs in any of the health-span or lifespan-related traits [30]. This indicates that we are only at the beginning of the longer journey to understand the mechanistic relevance of DNAm for our aging biology.

## Materials and methods

### Sample

All panel respondents who completed a Health and Retirement Study (HRS) interview during the 2016 wave and underwent a venous blood draw provided consent. The request was made by the HRS interviewer at the end of the interview (telephone and in person modes), with the offer of a 50-dollar incentive payment to be sent by check. That incentive amount and procedure is consistent with other voluntary ancillary activities in HRS, which is paid in advance of completion. No higher amounts were offered. The present study was approved by the Institutional Review Board at the University of Southern California (UP-20–00219).

### The HRS methylation sample

DNA methylation assays were completed on a representative sub-sample (*N* = 4018) of people who participated in the Health and Retirement 2016 Venous Blood Study[44]. Full details on the blood sample collection are provided in SI Appendix 1. As reported previously[45], the DNA methylation data are based on assays using the Infinium Methylation EPIC 1.0 BeadChip completed at the Advanced Research and Diagnostics Laboratory at the University of Minnesota. Samples were randomized across plates by key demographic variables (i.e., age, cohort, sex, education, race/ethnicity) with 39 pairs of blinded duplicates. Analysis of duplicate samples showed a correlation > 0.97 for all replicated CpG sites. The *minfi* package[46] in the R software was used for data pre-processing and quality control; 3.4% of the methylation probes (*n* = 29,431 out of 866,091) were removed from the final data set due to suboptimal performance (using a detection *p*-value threshold of 0.01). Analysis for detection *p*-value failed samples was done after removal of detection *p*-value failed probes. Using a 5% cutoff (*minfi*), 58 samples were removed. Sex-mismatched samples and any controls (cell lines, blinded duplicates) were also removed. For the present study, we used data assayed from only the EPIC 1.0 chip and removed CpG sites that were not in common between the EPIC 1.0 and EPIC 2.0 chips for greater future translatability across other cohorts. This resulted in 697,684 CpG sites for analyses. Methylation scores for each CpG site were represented as a beta value, calculated from the fluorescent intensity ratio, ranging from 0 (completely unmethylated site) to 1 (completely methylated).

### Biomarkers in the measured Physiological Health Risk Score (PhysRS) in the HRS

Four of the measures—*pulse pressure*, *peak flow*, *waist-to-height ratio*, and *HbA1c*—come from data collected in the home by interviewers, including dried blood spots (described below), in half of the samples in 2014 and half in 2016 [47, 48]. Systolic and diastolic blood pressure was assessed while seated and involved three measurements taken on the left arm 45 s apart using an Omron HEM-780 Intellisense Automated blood pressure monitor with ComFit cuff. The three measurements were averaged to derive values for SBP and DBP, and pulse pressure was calculated as SBP-DBP. Peak flow was assessed using a Mini-Wright peak flow meter with a disposable mouthpiece. Three readings were taken 30 s apart and averaged. The respondent’s height was measured in inches, to the nearest quarter inch, by the interviewer using a measuring tape and rafter’s square (details in SI Appendix 1).

*High sensitivity C-reactive protein* (hsCRP) was measured in serum using a latex-particle enhanced immunoturbidimetric assay kit (Roche Diagnostics, Indianapolis, IN) and read on the Roche COBAS 6000 Chemistry analyzer (Roche Diagnostics)[49]. The reference range is 0–5 mg/L. The laboratory inter-assay CV is 5.1% at a concentration of 1.05 mg/L and 6.7% at a concentration of 3.12 mg/L. High-density lipoprotein-cholesterol (HDL-C) was measured directly in serum using the Roche HDL-Cholesterol third-generation direct method (Roche Diagnostics) on the Roche COBAS 6000 analyzer. This method is standardized against the designated CDC reference method,; and calibration of the assay is regularly monitored by the CDC/NHLBI Lipid Standardization Program. The NCEP program recommends a reference range of > 40 mg/dL. The laboratory inter-assay CV is 1.3% at a mean concentration of 26.6 mg/dL and 1.3% at a mean concentration of 51.3 mg/dL. *Cystatin C* was measured in serum or plasma using cystatin C reagent (Gentian, Moss Norway) on the Roche COBAS 6000 analyzer. The laboratory inter-assay CV is 4.3% at a concentration of 0.75 mg/L and 3.2% at a concentration of 3.83 mg/L. *Dydroeplandrosterone sulfate* (DHEAS): DHEAS was measured in serum using the Roche e411 Analyzer using a competition immunoassay method (Roche Diagnostics). The laboratory inter-assay CV is 5.3% at 4.87 mol/L and 5.1% at 13.21 mol/L. *Glycosylated hemoglobin (HbA1c)* assay was performed on dried blood samples (DBS) using a Bio-Rad Laboratories Variant II High Pressure Liquid Chromatography (HPLC) System (Hercules, CA) optimized to accommodate the limited microliter volume available from a DBS sample. Principal reagents were the US Food and Drug Administration (FDA)-cleared Variant II Hemoglobin A1C Program (FDA K070452 and FDA K130860) obtained from Bio-Rad. DBS assay results were verified by comparing %HbA1c values obtained from 177 DBS samples versus DBS-matched blood samples (all samples analyzed on the Variant II). The correlation coefficient of the linear regression (LR) comparison was *R*^2^ = 0.99. Because HbA1c was not assayed in the 2016 venous blood collection but was one of the assays done using DBS collected in 2014 and 2016, HbA1c is based on the venous blood equivalent value associated with the DBS assay.

### Development and validation of DNAm Physiological health Age (PhysAge)

We used a two-stage process to develop and validate DNAm surrogates for eight biomarkers. First, in order to develop a separate training and test sample, the HRS methylation sample with complete data on biomarkers (*n* = 3177) was randomly split in half, *n* = 1589 training, and *n* = 1588 test sets. In step 1, we determined tuning parameters of alpha and lambda using *k*-fold cross validation [50] with *k* = 10. Briefly for this step, which has been described previously [51], the data were divided into 10 equivalent sized subsets, and for each value of lambda and for each subset, a linear prediction model biomarker was fitted to the data in *k* − 1 subsets. Then, the fitted model is used to estimate the prediction error in the subset that was left out. This was repeated for all *k* subsets, and results in an estimate of the prediction error for each lambda. This was completed at varying levels of alpha (0, 0.25, 0.5, 0.75, and 1). The penalty parameters of alpha and lambda were chosen to minimize prediction error, using the mean-squared error (MSE). In step 2, we used elastic net regularization to develop an algorithm with DNAm levels predicting each biomarker. The initial cross-validation and elastic net prediction were run in the 50% training sample at the development stage. CpGs identified and respective beta coefficients from the regularized prediction model were then used to calculate and validate the DNAm surrogate scores in the 50% test sample. In the test sample, each DNAm surrogate was calculated as a sum of the weighted values for each CpG, then standardized (mean = 0, sd = 1). We then calculated a sum of the eight DNAm surrogates, and the total score was standardized.

This produced a DNAm surrogate sum for PhysAge. For increased interpretability, we next re-calibrate the standardized DNAm PhysAge surrogate sum score to reflect the same mean and variance of age in the training data with the following:

$$\text{DNAm PhysAge}={(\mathrm{PhysAge}}_{\mathrm{surrogate}}* 9.6304)+ 68.1473$$

### Epigenetic clocks

We compare associations with health outcomes for DNAm PhysAge and three later-generation clocks—PhenoAge, GrimAge2, DunedinPACE—that have shown the strongest relationships with health outcomes [8, 52]. The PhenoAge clock [20] comprised 513 CpG sites that exhibited marked differences in disease and mortality among individuals of the same calendar age. GrimAge [7] was constructed with eight DNAm-based surrogate biomarkers (for pack years, adrenomedullin, beta-2 microglobulin, cystatin C, growth differentiation factor 15, leptin, plasminogen activation inhibitor, and tissue inhibitor metalloproteinase) across 1030 CpG sites that jointly predict mortality risk. The GrimAge2 clock includes all of the previously identified DNAm proteins but now also includes surrogates for CRP and Hba1c. We use this latter measure in the analysis as it was recently shown to outperform its progenitor in a meta-analysis involving data for 10,065 individuals and nine cohorts. The DunedinPACE [22] clock represents a rate measure (i.e., how fast a person is aging) compared with a state measure (i.e., how much aging has occurred up to that point) and was developed by identifying DNAm correlates (173 CpG sites), at a single time point, of decline in 19 indicators of organ-system integrity across four measurement occasions spanning two decades. Further comparisons on development of these clocks is provided in SI Appendix 1.

## Health outcomes

We use multiple aging phenotypes to evaluate prediction from PhysAge and surrogates, an approach originating from prior studies [53, 54], to include physical functioning, cognitive functioning, and a subjective health measure.

### Walking speed

Walking speed is an important objective indicator of vitality in mid-life and older ages [55]. Walking speed was measured by marking a course of 98.5 inches (250 cm) in a suitable space in the respondent’s home with a tape measure and placing masking tape at the starting and ending points. The interviewer used a stopwatch to record the time from which the respondent’s foot first crossed the starting point and touched the floor and the time their foot touched the floor after fully crossing the ending point. The respondent was asked to complete two timed walks (to one end, stop, and back). Walking speed was conducted with respondents aged 65 years or older in HRS, and the average of two-timed walks was calculated.

### Maximal grip strength

Grip strength has prognostic value as an indicator of functional decline and mortality at older ages [56]. Participants were instructed to squeeze the Smedley spring-type hand dynamometer with maximal effort and then let go. Assessments were administered with participants standing with their arm at their side and with the elbow flexed at a 90-degree angle. After one practice trial, two measurements were taken for each hand, alternating hands. The maximal reading from the four trials was used to indicate maximal grip strength capacity.

### Frailty

The HRS Frailty Index (FI) [57] consists of 39 measures including difficulty with activities of daily living (walking, transferring, bathing, dressing, eating, toileting), instrumental activities of daily living (making a phone call, managing money, taking medications, shopping, preparing meals), memory (immediate and delayed word recall), psychiatric problems, joint replacement, difficulty with vision or hearing, and difficulty with Nagi functioning (using a map, yard work, walking one block, sitting, getting up from chair, climbing several flights of stairs without resting, climbing one flight of stairs without resting, stooping/kneeling/crouching, reaching arms above shoulder level, pushing or pulling large objects, picking up a dime, lifting or carrying weights over 10 pounds, self-rated health), and the presence of chronic diseases (i.e., hypertension, angina, congestive heart failure, diabetes, stroke, lung disease, arthritis, cancer, heart attack). Binary variables were coded as 0 or 1 to indicate the absence or presence of deficits. Continuous and ordinal variables were categorized from 0 to 1 to indicate various degrees of deficits. Then, the sum of deficit scores was divided by the total number of measures and multiplied by 100.

### Disability (ADLs and IADLs)

Limitations in activities of daily living (ADLs) and instrumental activities of daily living (IADLs) are included as a proxy for the participant’s general physical condition. ADLs included difficulties with (1) dressing, (2) walking across a room, (3) bathing or showering, (4) eating, such as cutting up food, (5) getting in or out of bed. IADLs included (1) difficulties in preparing a hot meal, (2) shopping for groceries, (3) making telephone calls, (4) taking medications, and (5) managing money. We summed the number of conditions separately with respect to ADLs and IADLs, and the count of these conditions (range = 0–5) is used in the analysis.

### Self-rated health

Participants were asked to rate self-rate their health on a 5-point rating scale: *Excellent*, *Very Good*, *Good*, *Fair*, or *Poor*. Responses were subsequently recoded to produce a binary measure by comparing those reporting excellent/very good/good health with those reporting fair/poor health.

### Errors in cognition tests

The score indicates the number of errors on the Telephone Interview of Cognitive Status Exam (TICS)[58], including immediate recall, delayed recall, serial 7 s, and backwards counting tasks, which ranges from 0 to 27. For immediate recall, interviewers read a list of 10 words, and participants were asked to recall as many as possible (range 0–10). For delayed recall, participants were asked to recall the same 10 words after about 5 min (range 0–10). For the serial 7 s task, participants were asked to count backwards from 100 by 7 for five trials (e.g., 93, 86, 79, 72, 65; range 0–5). For the backwards counting task, participants were asked to count backwards from 20 by 1 for 10 continuous numbers, scoring 0 errors if they did it successfully on two trials, 1 error if they did it successfully on one trial, and 2 errors if successful on neither trial.

### Mortality

Mortality was coded as 0 for still living or as 1 for 288 of 1588 participants that died in the follow-up period from the time when participants provided a blood sample through to the 2022 follow-up survey. The range of survival months was 0 to 90 months, with a mean of 63.53 months (SD = 17.46).

### Statistical analysis

The analyses were conducted using R 4.3.1 (R Foundation for Statistical Computing, Vienna, Austria) or Stata 18.0 (StataCorp, College Station, TX). The outcome measures were regressed separately on each of the eight DNAm surrogates and five age residualized biological ageing measures (PhysRS, PhysAge, GrimAge2, PhenoAge, and DunedinPACE) adjusting for chronological age (years), sex, ethnicity, and WBC counts using ordinary least squares regression (walking speed, grip strength, frailty, TICS errors in HRS), Poisson regression (disability scores), negative binomial regression (MOCA errors in TILDA and NICOLA), logistic regression (binary self-reported health), and Cox regression (mortality) as appropriate. We report the change in each of the outcome measures associated with a standard unit increase in each of the standardized age residualized biological aging measures. In the analyses involving walking speed [or TUG] and grip strength, we adjusted additionally for measured height (cm), which differs between men and women and is strongly associated with stride length and strength. When calculating the area under the receiver operating curve (AUC) for each outcome, we first dichotomized each continuous phenotype by using the median value, and then ran a logistic regression and calculated the AUC using the pROC package in R (for HRS and NICOLA) or roccomp command in STATA (for TILDA). Because HRS does not allow publication of individual-level data, to present profiles of individuals with PhysAge and DNAm surrogates in metrics of age, we simulated data for DNAm surrogate profiles. Simulated data were generated separately for men and women using the mvnorm command in the R MASS package in order to match mean, standard deviation, distribution, and correlation structure of the HRS test set. Gene trait enrichment analysis was performed by identifying coding gene sets that mapped to CpG surrogate sets as a composite and individually, then using StringDB *v*.12.0 to determine whether gene sets implicated functional properties (details provided in SI Appendix 1).

## Supplementary Information

Below is the link to the electronic supplementary material.

Supplementary file1 (XLSX 6.84 MB)

Supplementary file2 (DOCX 6.40 MB)

## Data Availability

Anonymized HRS data have been deposited at the University of Michigan, HRS website (https://hrs.isr.umich.edu). DNA methylation data have been deposited into the NIAGADS Data Sharing Service (https://dss.niagads.org), and researchers must apply for data access. For TILDA, researchers must apply to use a hot desk facility (https://tilda.tcd.ie/data/accessing-data/) to analyze data. Access to NICOLA data is available for bona fide researchers through application (https://www.qub.ac.uk/sites/NICOLA/InformationforResearchers/#research-proposals-approved-910951-2).

## Declarations

### Code for analysis

Code used for analyses and to calculate the DNAm PhysAge score and DNAm surrogates will be accessible via GitHub (https://github.com/em-arpy) upon article publication.

### Epigenetic clocks

Epigenetic clock and age acceleration measures for PhenoAge used in these analyses are available on the HRS website for download by registered users (https://hrsdata.isr.umich.edu/data-products/epigenetic-clocks) or can be calculated from DNA methylation data using code for GrimAge2 (requested from Drs. Steve Horvath or Ake Lu) and DunedinPACE (https://github.com/danbelsky/DunedinPACE).
