# Supplementary material for: Physiological health Age (PhysAge): a novel multi-system molecular timepiece predicts health and mortality in older adults
Source: GeroScience. 2025 Sep 1;48(2):3115–35. doi: 10.1007/s11357-025-01832-1 (PMC12972485; doi:10.1007/s11357-025-01832-1)
Supplement: Supplementary file 2 — Supplementary file2 (DOCX 6.40 MB) [file 11357_2025_1832_MOESM2_ESM.docx]

**Supporting Information for**

Physiological Health Age (PhysAge): A novel multi-system molecular timepiece predicts health and mortality in older adults

Author list: Thalida Em Arpawong, Belinda Hernandez, Claire Potter, Robert J. Leigh, Eric T. Klopack, Claire Hill, Giovanni Fiorito, Laura J. Smyth, Aisling M. O’Halloran, Bernadette McGuinness, Jessica D. Faul, Rose Anne Kenny, Amy Jayne McKnight, Eileen M. Crimmins, Cathal McCrory

Corresponding author: Thalida Em Arpawong

Email: [arpawong@usc.edu](mailto:arpawong@usc.edu)

**This PDF file includes:**

Supporting text

Figures S1 to S11

Tables S1 to S5

SI References

Table of Contents

[Epigenetic Clocks used for comparison of DNAm PhysAge 4](#_Toc191491244)

[The US Health and Retirement Study (HRS) 5](#_Toc191491245)

[HRS Blood Sample Collection 5](#_Toc191491246)

[HRS Height measurement 5](#_Toc191491247)

[The Irish Longitudinal Study on Ageing (TILDA) 6](#_Toc191491248)

[The TILDA epigenetic sample 6](#_Toc191491249)

[The Northern Ireland Cohort for the Longitudinal Study of Ageing (NICOLA) 7](#_Toc191491250)

[The NICOLA epigenetic sample 7](#_Toc191491251)

[MEASUREMENT OF HEALTH OUTCOMES IN TILDA and NICOLA 7](#_Toc191491252)

[Walking speed / Timed-Up-and-Go 8](#_Toc191491253)

[Maximal Grip Strength 8](#_Toc191491254)

[Frailty index 8](#_Toc191491255)

[Limitations in activities of daily living (ADLs) and instrumental activities of daily living (IADLs) 8](#_Toc191491256)

[Montreal Cognitive Assessment (MOCA) 9](#_Toc191491257)

[Self-rated health 9](#_Toc191491258)

[Mortality 9](#_Toc191491259)

[Gene trait enrichment analysis 11](#_Toc191491260)

[Gene-set interaction network visualisation 12](#_Toc191491261)

[Supplementary Figures 13](#_Toc191491262)

[Figure S1a. Pearson correlations of measured values vs DNA methylation surrogate biomarkers in the HRS test set (n=1588). 13](#_Toc191491263)

[Figure S1b. Correlations of measured values vs DNA methylation surrogate markers in the TILDA validation sample (n=488) 14](#_Toc191491264)

[Figure S1c. Correlations of measured values vs DNA methylation surrogate markers in the NICOLA validation sample (n=1,830) 15](#_Toc191491265)

[Figure S2a. Comparing prediction of aging phenotypes from six DNAm surrogates and measured biomarkers in TILDA. 16](#_Toc191491266)

[Figure S2b. Comparing prediction of aging phenotypes from six DNAm surrogates and measured biomarkers in NICOLA. 17](#_Toc191491267)

[Figure S3a. Intercorrelations of the different biological ageing measures (TILDA) 18](#_Toc191491268)

[Figure S3b. Intercorrelations of the different biological ageing measures (NICOLA) 19](#_Toc191491269)

[Figure S4a. Associations of the individual DNA methylation surrogate biomarkers comprising the DNAm PhysAge with a range of clinical phenotypes in TILDA. 20](#_Toc191491270)

[Figure S4b. Associations of the individual DNA methylation surrogate biomarkers comprising the DNAm PhysAge with a range of clinical phenotypes in NICOLA. 21](#_Toc191491271)

[Figure S5a. Comparison of the prediction for aging phenotypes from the physiological health risk score (PhysRS) and Epigenetic Clocks in HRS. 23](#_Toc191491272)

[Figure S5b. Comparison of the prediction for aging phenotypes from the physiological health risk score (PhysRS) and Epigenetic Clocks in TILDA. 24](#_Toc191491273)

[Figure S5c. Comparison of the prediction for aging phenotypes from the physiological health risk score (PhysRS) and Epigenetic Clocks in NICOLA. 26](#_Toc191491274)

[Figure S6. Comparisons of the DNAm surrogates in common between PhysAge and GrimAge in the prediction of aging phenotypes in the HRS test sample (n=1588). 27](#_Toc191491275)

[Figure S7. Gene network enrichment analysis of the DNAm surrogate sites comprising the DNA methylation Physiological health age (PhysAge) score. 28](#_Toc191491276)

[Figure S8. Jaccard indices between gene sets indicate the overall shared content observed between gene sets, expressed as a proportion. 29](#_Toc191491277)

[Figure S9. Venn Diagram describes the number of overlapping CpG sites in the 2^nd^ generation clocks. 30](#_Toc191491278)

[Figure S10. Heatmap of correlations between DNAm surrogates and their clinical biomarker in the HRS test set (n=1588). 31](#_Toc191491279)

[Figure S11. Heatmap of correlations between DNAm surrogates and health assessments in the HRS test set (n=1588). 32](#_Toc191491280)

[SUPPLEMENTARY TABLES 33](#_Toc191491281)

[Table S1a. Summary statistics for the biological ageing measures and health outcomes in the TILDA validation sample (n=488) 33](#_Toc191491282)

[Table S1b. Summary statistics for the biological ageing measures and health outcomes in the NICOLA validation sample (n=1,830) 34](#_Toc191491283)

[Table S2a. Summary Measures and Area Under the Curve (AUC) for HRS 35](#_Toc191491284)

[Table S2b. Summary Measures and Area Under the Curve (AUC) for TILDA 37](#_Toc191491285)

[Table S2c. Summary Measures and Area Under the Curve (AUC) for NICOLA 39](#_Toc191491286)

[Table S3. Comparisons of DNAm surrogates used to develop both PhysAge and GrimAge2 41](#_Toc191491287)

[Table S4. String DB links for coding gene sets that mapped back to CpG surrogate sets 42](#_Toc191491288)

[Table S5. Simulations of DNAm surrogates with PhysAge of 60 for four men and PhysAge of 80 for four women 43](#_Toc191491289)

[SI References 44](#_Toc191491290)

# **Epigenetic Clocks used for comparison of DNAm PhysAge**

The PhenoAge clock(1) was developed using a 2-stage approach. In the first stage, a weighted composite of 10 clinical characteristics (calendar age, albumin, creatinine, glucose, C-reactive protein, lymphocyte percentage, mean cell volume, red blood cell distribution weight, alkaline phosphatase, and white blood cell [WBC] count) was used to develop a phenotypic age estimator. In the second stage, the phenotypic age estimator was regressed on DNAm levels resulting in the identification of 513 CpG sites that exhibited marked differences in disease and mortality among individuals of the same calendar age.

GrimAge(2) was also developed using a 2-stage process. In the first stage, Lu and colleagues regressed levels of each of 88 plasma proteins and smoking pack years on chronological age, sex, and [CpG](https://www.sciencedirect.com/topics/medicine-and-dentistry/guanine) levels, identifying CpG sites whose linear combination best predicted corresponding plasma protein levels in the training dataset. They identified 12 DNAm based surrogate biomarkers with a Pearson correlation coefficient higher than 0.35 with measured levels of the biomarker in the training and test datasets. In the second stage, they regressed time to death due to all-cause mortality on age, sex, DNAm pack years, and the 12 DNAm surrogate biomarkers of plasma protein levels, with the elastic net selecting 8 DNAm based biomarkers (DNAm pack years DNAm adrenomedullin, DNAm beta-2 microglobulin, DNAm Cystatin C, DNAm growth differentiation factor 15, DNAm leptin, DNAm plasminogen activation inhibitor, and DNAm tissue inhibitor metalloproteinase) across 1030 CpG sites that jointly predicted mortality risk. The GrimAge2 clock includes all of the previously identified DNAm proteins but now also includes surrogates for CRP and Hba1c. We use this latter measure in the analysis as it was recently shown to outperform its progenitor in a meta-analysis involving data for 10,065 individuals and nine cohorts.

The DunedinPACE(3) clock is qualitatively different from other aging clocks as it represents a rate measure (i.e. how fast a person is aging) compared with a state measure (i.e. how much aging has occurred up to that point) and was developed by identifying DNAm correlates (173 CpG sites), at a single time point, of decline in 19 indicators (body mass index, waist-hip ratio, glycated haemoglobin, leptin, forced vital capacity ratio (FEV_1_/FVC) , forced expiratory volume in 1 second (FEV1), total cholesterol, triglycerides, high density lipoprotein, lipoprotein(a), apolipoprotein B100/A1 ratio, estimated glomerular filtration rate, blood urea nitrogen, high sensitivity CRP, white blood cell count, mean periodontal attachment loss, number of dental caries affected tooth surfaces) of organ-system integrity across four measurement occasions spanning two decades.

# **The US Health and Retirement Study (HRS)**

**HRS Blood Sample Collection**

The blood collection was managed by Hooper Holmes Health & Wellness. The phlebotomy service was provided with the names, addresses, and phone numbers of consenting respondents and contacted respondents to set appointments. Collection materials were mailed to the phlebotomists’ homes in advance of the scheduled visit. Every attempt was made to schedule the blood draw within 4 weeks of the HRS core interview. Fasting was recommended and preferred but not required. Phlebotomists noted the fasting status of the samples. 50.5 mL of blood was collected in six tubes – one 8 mL CPT tube, three 10 mL double gel serum separator tubes (SST), one 10 mL EDTA whole blood tube, and a 2.5 mL PAXgene RNA tube. The SST tubes were centrifuged in the field before being shipped overnight to the CLIA-certified Advanced Research and Diagnostic Laboratory at the University of Minnesota. Tube processing was done within 24 hours of arrival at the lab (within 48 hours of collection)(4).

**HRS Height measurement**

Height was assessed using a tape measure and rafter’s square. Respondents were requested to remove their shoes and stand with their heels and shoulders against the wall. An adhesive note was placed just behind the participant’s head. The interviewer placed the rafter’s square on the respondent’s head and parallel against the wall and marked the adhesive note at the bottom of the rafter’s square. The interviewer then asked the respondent to move away from the wall and used the tape measure to determine the respondent’s height by measuring from the floor up the mark on the adhesive note. Respondents were requested to remove their shoes and any bulky clothing. The respondent’s height was recorded in inches to the nearest quarter inch.

# **The Irish Longitudinal Study on Ageing (TILDA)**

The TILDA study involves a nationally representative sample of 8175 community-dwelling older persons aged 50 years and older at baseline. Respondents complete a 90-minute computer-assisted personal interview in the home and a separate self-completion questionnaire containing more sensitive information that is returned via post. All participating respondents are invited to attend for a detailed clinic-based health assessment administered by trained nursing staff in either a center-based or home-based setting. Ethical approval for the Irish Longitudinal Study on Ageing (TILDA) study was obtained from the Trinity College Dublin Research Ethics Committee and signed informed consent was obtained from all participants.

## **The TILDA epigenetic sample**

The present study uses a subsample (n = 490) of the baseline TILDA cohort who were selected into the epigenetic study based on their life course social class trajectory into 4 groups: stable high, stable low, upwardly mobile, downwardly mobile as described in(5). DNAm levels were assessed using the Infinium Human Methylation 850k EPIC Beadchip (Illumina Inc., San Diego, CA). Regarding data preprocessing TILDA performed a harmonised processing procedure to that of the HRS and NICOLA studies to ensure that differences observed between cohorts were not due to biases or variability in the data processing pipeline. Hence as described in the methods section for HRS, TILDA pre-processing and quality control was performed using the minfi package in R. In total n=488 samples were included in the current analysis. Of the 10 samples which were excluded from analysis: 5 were found to have sex discordance, 1 failed the Illumina bisulfite conversion QC check and another 4 were removed due to two sets of duplications. A total 116,154 probes were deemed unreliable and removed from the analysis due to having a detection p-value>0.01 and/or <4 hybridizing beads in more than 5% of the samples leaving a total of 749,705 for analysis. As in HRS NOOB normalisation was performed.

# **The Northern Ireland Cohort for the Longitudinal Study of Ageing (NICOLA)**

The NICOLA study recruited 8,283 community-dwelling older persons aged 50 years and older from a randomised sample of Northern Ireland (NI) addresses obtained from the Business Service Organization General Practitioner Register stratified by geographical location and postcode (6). If a spouse or partner also resided in the home at time of interview, regardless if aged over 50 they were also invited to participate (n = 195) giving a final cohort size of 8,478. All participants underwent a computer-assisted personal interview, invited to return a self-completion questionnaire and were invited to attend for a detailed clinic-based health assessment in either a center-based or home-based setting. The NICOLA study underwent ethical review, approval and is sponsored by the School of Medicine, Dentistry and Biomedical Sciences of Queen’s University Belfast (Ref: 12/23). Participants provided written informed consent prior to participation in the study.

## **The NICOLA epigenetic sample**

The first 2,000 samples that had sufficient high quality DNA extracted from buffy coats of whole blood of participants that attended NICOLA's health assessment were selected for DNA methylation (DNAm)(7). DNAm status was determined using the Infinium MethylationEPIC BeadChip array (Illumina Inc., San Diego, CA) following the manufacturer’s instructions and has been described in detail elsewhere(8). Eight samples were unsuitable and used as duplicates and 16 participants have subsequently removed consent and requested their data be removed. 106 samples were lost during the quality control (QC) process using the minfi package in R in the same pipeline as HRS and TILDA. Samples of a further 40 participants aged under 50 years were excluded from this analysis giving a final DNAm sample size for NICOLA of 1,830 (22.1% of NICOLA cohort). All participants in the NICOLA DNAm cohort are of white of European ancestry(9) and so adjustment in models for race/ ethnicity was not completed.

# **MEASUREMENT OF HEALTH OUTCOMES IN TILDA and NICOLA**

## **Walking speed / Timed-Up-and-Go**

Walking speed was assessed using a 4.88 meter (m) computerized walkway with embedded pressure sensors (GAITRite, CIR Systems Inc., New York, NY). Participants completed 2 walks along the mat at their normal walking speed. Each trial started 2.5 m before and ended 2 m after the walkway in order to allow room for acceleration and deceleration. The average of the 2 readings represented the overall walking speed measure expressed in centimeters traveled per second (cm/s). In NICOLA, walking speed was not captured in the Wave 1 Health Assessment. Instead, the timed up-and-go (TUG) test of mobility is assessed. The TUG indicates the time taken by a participant to stand from a standard arm chair, walk three meters at usual pace, turn, walk back to the chair and sit down(10).

## **Maximal Grip Strength**

Grip strength was measured using a Baseline Hydraulic Hand Dynamometer, which consisted of a gripping handle with a strain gauge and an analogue reading scale that increased in 2-kilogram (kg) increments. Respondents were instructed to hold the device in their hand with the forearm at a right angle to their upper arm. The participant was then instructed to squeeze as hard as they could for a few seconds. Results were rounded to the nearest whole number (ie, if the dial fell between 22 and 24 kg, grip strength was recorded as 23 kg). This procedure was repeated twice in each of the dominant and nondominant hands and the maximum of the 4 measurements was used to indicate grip strength.

## **Frailty index**

Frailty was operationalized using a modified 40-item version of Rockwood’s frailty index(11) adapted to the TILDA database and 30-item version adapted to the NICOLA database(12). It measures the number of health deficits present as a proportion of the total number of potential health deficits. The deficits included any symptom, sign, disease, disability, associated with age and adverse outcomes. The outcome measure was log transformed prior to analysis.

## **Limitations in activities of daily living (ADLs) and instrumental activities of daily living (IADLs)**

Limitations in activities of daily living (ADLs) and instrumental activities of daily living (IADLs) are included as a proxy for the participant’s general physical condition. ADLs included difficulties with (1) dressing, (2) walking across a room, (3) bathing or showering, (4) eating, such as cutting up food, (5) getting in or out of bed, and (6) using the toilet. IADLs included (1) difficulties in preparing a hot meal, (2) doing household chores, (3) shopping for groceries, (4) making telephone calls, (5) taking medications, and (6) managing money. We summed the number of conditions separately with respect to ADLs and IADLs, and the count of these conditions is used in the analysis.

## **Montreal Cognitive Assessment (MOCA)**

Respondents’ cognitive status was measured using the Montreal Cognitive Assessment (MOCA) (Nasreddine et al, 2005). The MOCA was designed as a brief cognitive screening tool to detect mild cognitive impairment (MCI) among community and clinic-based samples. The test takes approximately 10 minutes to administer and assesses performance across a number of cognitive domains including: *executive functioning* (e.g. alternating letter number trail making task); *visuospatial abilities* (e.g. clock drawing); *attention/concentration* (e.g. target detection using tapping); *language* (e.g. verbal fluency task), *short term memory* (e.g. immediate and delayed word recall); and *orientation* (e.g. current date/time/year). The instrument yields a sum score ranging from 0-30. We calculated the number of errors by subtracting the number of incorrect responses from the total score and used this as the dependent variables in the analysis.

## **Self-rated health**

Participants were asked to rate self-rate their health on a 5-point rating scale: *Excellent*, *Very Good*, *Good*, *Fair* or *Poor*. Responses were subsequently recoded to produce a binary measure by comparing those reporting excellent/very good/good health with those reporting fair/poor health.

## **Mortality**

In Ireland, all deaths are registered through the General Register Office (GRO). Mortality status and cause of death were determined by inspection of death certificates over a ~12-year follow-up using data linkage between TILDA and the GRO. The methodology and procedure for determining cause of death has been documented elsewhere (13). Data are available for all reported deaths of TILDA participants up to 31st January 2022. There was a total of 52 deaths. The mean follow-up time from baseline to death or censoring was 11.1 years ranging from 1.37-12.3 years in total. In NI, mortality status was generated for NICOLA participants based on data linkage with the NI Health and Social Care Business Services Organisation (BSO) Honest Broker Service(6). Data on the 161 reported deaths of NICOLA participants up to 1^st^ March 2023 were included. The mean follow-up time from date the participant had their blood drawn as part of their Health Assessment attendance to death or censoring was 7.83 years (SD 0.57).

# **Gene trait enrichment analysis**

This analysis answers the question of whether a given gene set (regardless of length) has functional properties at higher proportions than expected by chance. Coding gene sets that mapped back to CpG surrogate sets (*n*_cohorts_ = 8) were assessed individually (45 ≤ *n*_genes_ ≤ 337) and as a composite (*n*_genes_ = 1089) for trait enrichment using StringDB *v*.12.0(14) and the default Homo sapiens subsp. *sapiens* (taxid: 9606) background genome for statistical comparison. Mapping CpG sites to genes, regardless of the number of CpG sites represented by that gene (Table S4) was done to minimize the potential issue of multi-gene bias. FDR corrected *P*-values ≤ 0.05 for StringDB genes were considered statistically significant (Dataset S1A & Table S4). Note, for protein-protein interaction analysis, only “experiments”, “databases”, “co‑expression”, “neighborhood”, and “co‑occurrence” interactions with high confidence (interaction score ≥ 0.7) were considered (Dataset S1B & S1C). To assess gene set overlap between DNAm surrogate sets, Jaccard indices between gene sets were calculated (details below).

When considered individually, each DNAm surrogate set displayed highly different trait enrichments (Dataset S1A & Table S4). The CRP surrogate set was enriched for the suntan and nevus count human phenotypes, but no specific gene function. The DHEAS surrogate was only enriched for the non-small cell lung cancer KEGG pathway (hsa05223). The Cystatin-C surrogate set was enriched for signal regulatory elements and was considerably enriched for genes that are expressed in several tissues (“whole body” expression) with 131 of 160 genes annotated; furthermore, this gene set was considerably enriched (113 of 160 genes) for alternative splicing (KW-0025). The HbA1c surrogate set was significantly enriched for heterocyclic organic compound binding and, like Cystatin-C surrogates, was considerably enriched for whole body expression (136 of 166 genes) and alternative splicing (124 of 166 genes). The HDL-C surrogate set was significantly enriched for several haematological phenotypes, considerably enriched for phosphoproteins (KW-0597; 181 of 337) and for alternative splicing (218 of 337 genes). The Peak flow surrogate set was enriched for STAT3 signalling pathway regulation (WP4538) and for genes associated with X-linked monogenic/dominant disease. The pulse pressure surrogate cohort was not significantly enriched for any gene function, phenotype, or disease association. Finally, the waist-to-height (WHR) ratio surrogate set was significantly enriched for phosphoproteins (79 of 136 genes).

## **Gene-set interaction network visualisation**

A gene interaction network was derived using the interaction scores from public databases as described. A node represents a gene, and an edge represents an interaction between two genes. The edge list for the full StringDB network was downloaded for further processing without further shell interactors (i.e. genes not associated with a surrogate but determined to be interactors in StringDB). Genes that did not have an interaction partner were excluded from further processing to construct a clear image (Figure S7). Due to the enrichment and overlap of “alternative splicing”, “signalling”, and “phosphoproteins”, genes were assigned one of eight categories (seven categories from the three enrichments (alternative splicing does not appear as a single function) and an “other” category) and converted to a nodes list. The nodes list and edge list were concatenated to a gdf file and visualised using Gephi *v*.0.9.2(15), with the layout determined by the proportional Yifan Hu algorithm and default parameters (<https://cir.nii.ac.jp/crid/1370004237453048078>).

To assess gene set overlap between DNAm surrogate sets, genes were treated as binary operators (incidences), and Jaccard indices (*J*) between gene sets were calculated using the formula:

$$\text{J}_{\text{(A, B)}}\text{ =}\left( \frac{\text{A∩}\text{B}}{\text{A}\text{∪}\text{B}} \right)$$

As 0 ≤ *J* ≤ 1, similarity is apparent as *J →* 1 and, vice versa, dissimilarity is apparent as *J →* 0 (Figure S8).

While considerable functional overlap was observed between gene sets, the maximal Jaccard distance (the overall shared content expressed as a proportion) between two sets was approximately *J* = 0.044 suggesting that the most related sets only had an overlap of approximately 4.4% (Figure S8).

# **Supplementary Figures**


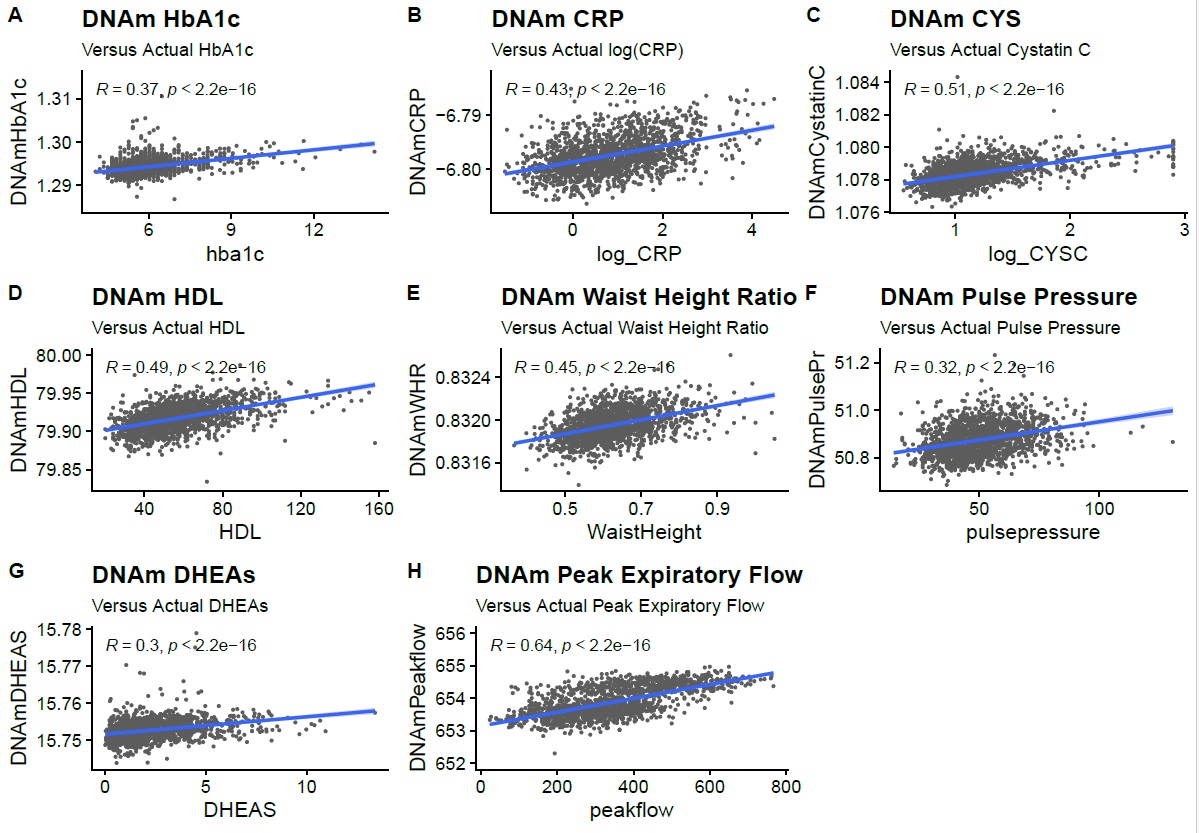


## **Figure S1a. Pearson correlations of measured values vs DNA methylation surrogate biomarkers in the HRS test set (n=1588).**


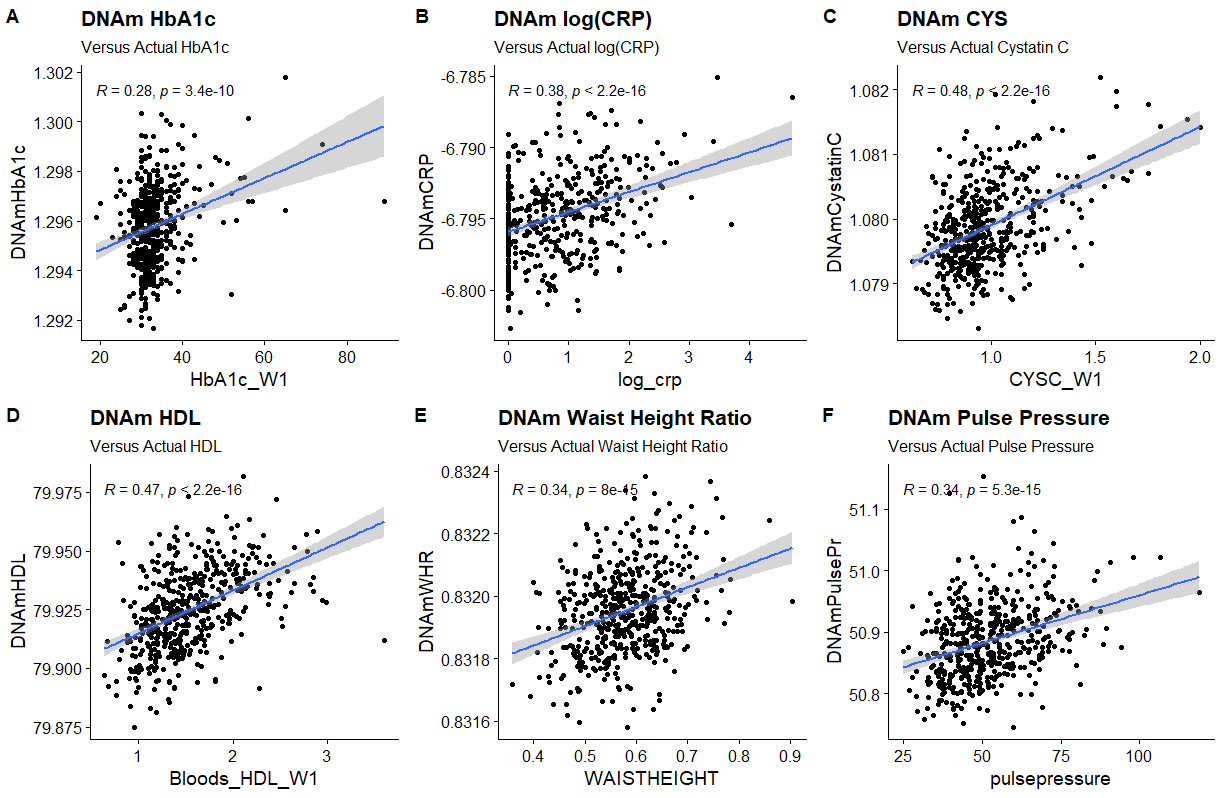


## **Figure S1b.** **Correlations of measured values vs DNA methylation surrogate markers in the TILDA validation sample (n=488)**

**
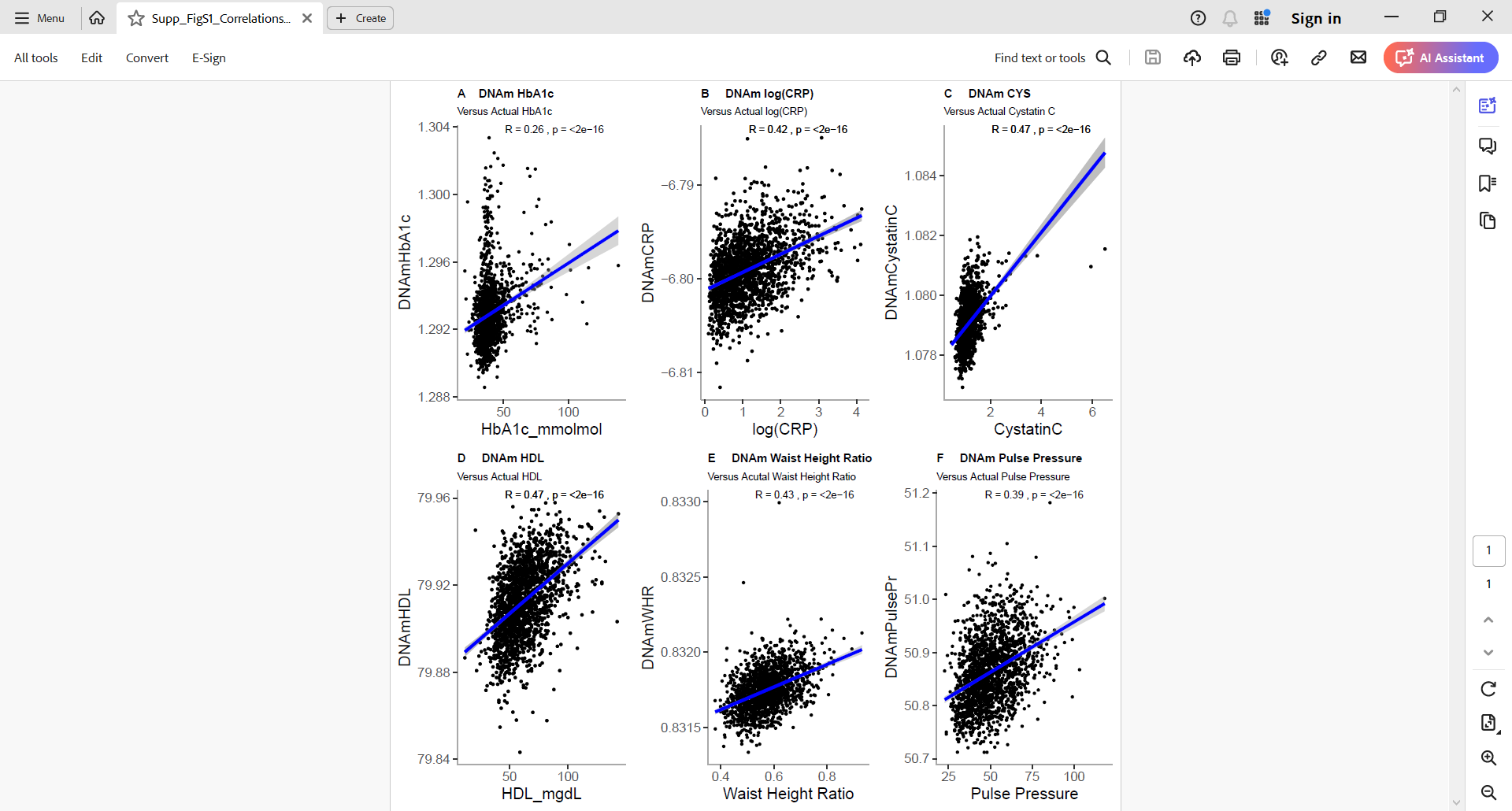
**

## **Figure S1c.** **Correlations of measured values vs DNA methylation surrogate markers in the NICOLA validation sample (n=1,830)**

## **Figure S2a. Comparing prediction of aging phenotypes from six DNAm surrogates and measured biomarkers in TILDA.**


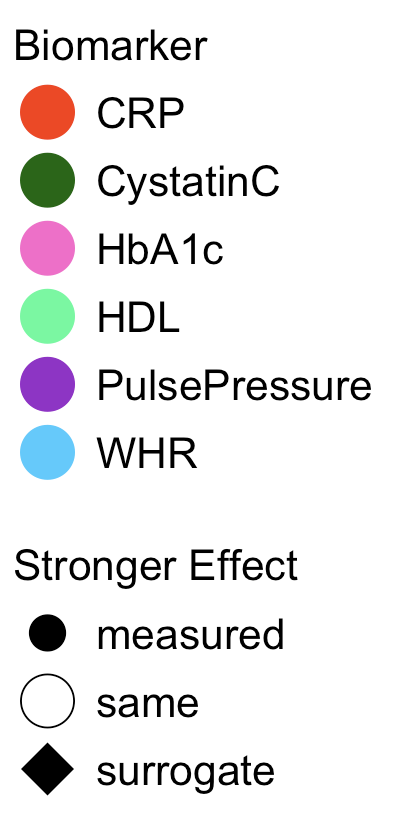

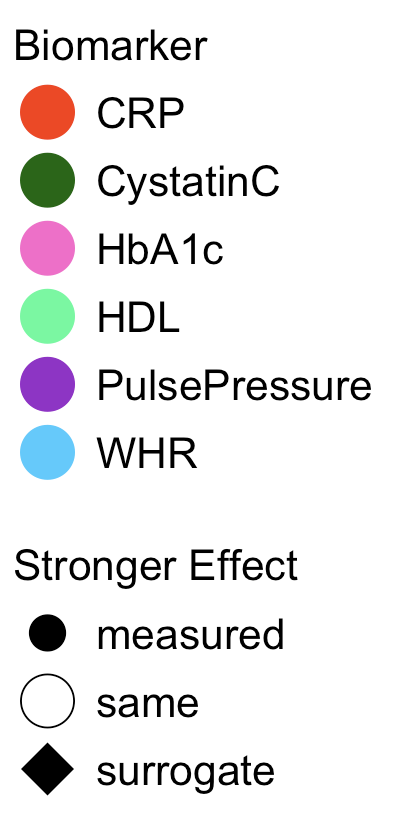


Each of the eight panels focuses on one of the aging phenotypes and shows standardized effect sizes for phenotype prediction from both the measured biomarker (x-axis) and the biomarker DNAm surrogate (y-axis). The gray diagonal line shows where X=Y and thus prediction from measured and DNAm biomarker would be equivalent. Biomarkers are indicated by a specific color and a filled diamond where prediction from the surrogate is stronger than the measured biomarker in the expected direction of effect, or a filled circle where the measured biomarker is stronger and a hollow circle where the effects of both are the same. The scales for HDL surrogate and biomarker are reversed whereby higher values are expected to correlate positively with the outcomes. All models were run with each DNAm surrogate or biomarker separately on the outcome, adjusted for age, sex, and race/ethnicity. Models for gait speed and grip strength were also adjusted for height.


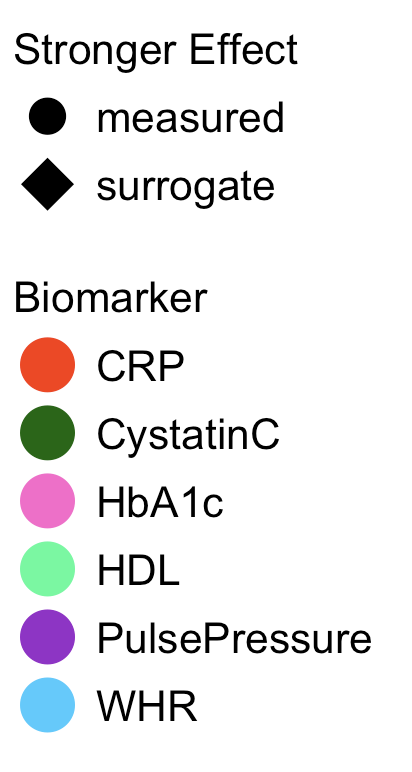


## **Figure S2b. Comparing prediction of aging phenotypes from six DNAm surrogates and measured biomarkers in NICOLA.**

Each of the eight panels focuses on one of the aging phenotypes and shows standardized effect sizes for phenotype prediction from both the measured biomarker (x-axis) and the biomarker DNAm surrogate (y-axis). The gray diagonal line shows where X=Y and thus prediction from measured and DNAm biomarker would be equivalent. Biomarkers are indicated by a specific color and a filled diamond where prediction from the surrogate is stronger than the measured biomarker in the expected direction of effect, or a filled circle where the measured biomarker is stronger and a hollow circle where the effects of both are the same. The scales for HDL surrogate and biomarker are reversed whereby higher values are expected to correlate positively with the outcomes. All models were run with each DNAm surrogate or biomarker separately on the outcome, adjusted for age, sex, and race/ethnicity. Models for gait speed and grip strength were also adjusted for height.

**
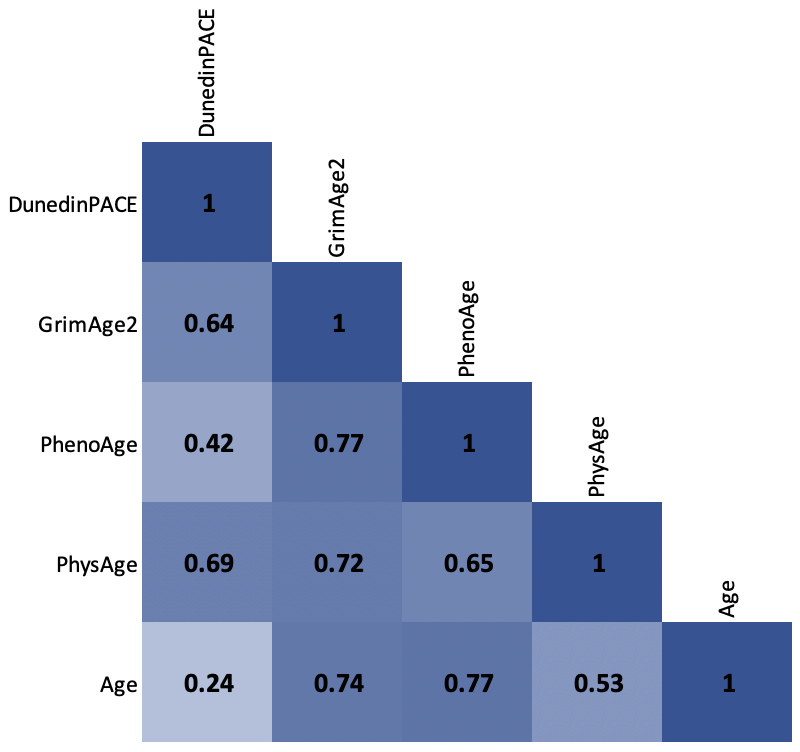
**

## **Figure S3a. Intercorrelations of the different biological ageing measures (TILDA)**

**
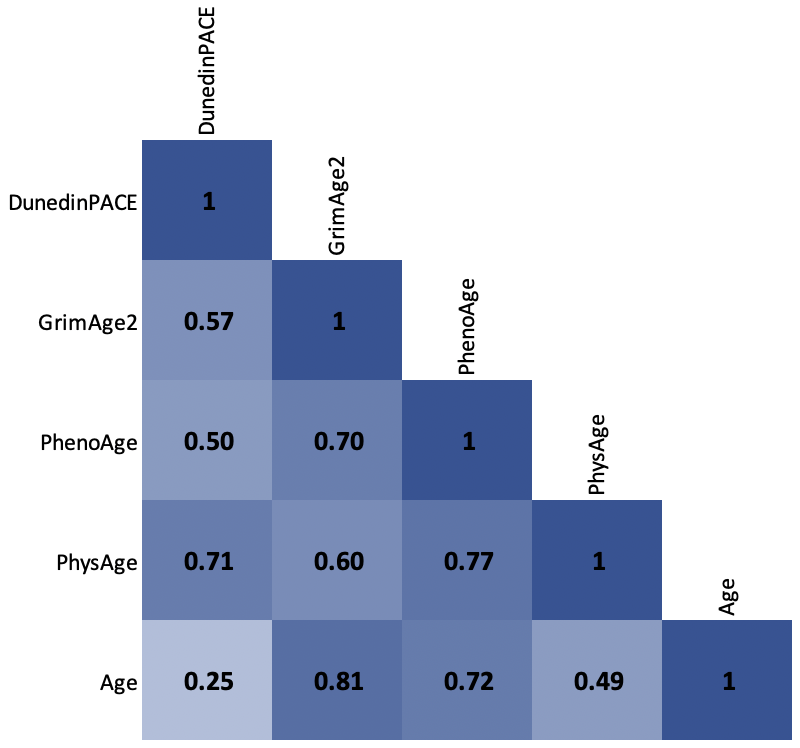
**

## **Figure S3b. Intercorrelations of the different biological ageing measures (NICOLA)**

## **Figure S4a. Associations of the individual DNA methylation surrogate biomarkers comprising the DNAm PhysAge with a range of clinical phenotypes in TILDA**.

Associations are adjusted for age, sex, and white blood cell counts. Effect sizes are displayed as beta values for walking speed, grip strength, and frailty; incident rate ratios for cognitive errors, ADLs, IADLs, and Self-Rated Health; and Hazard Ratios for Mortality. Scales for DNAm surrogates of HDL, DHEAS, and peak flow are reversed so that all surrogates are expected to show the same direction of effect with the phenotypes. All associations are adjusted for age, sex, and white blood cell counts. Associations with walking speed and grip strength were also adjusted for height.

********

## **Figure S4b. Associations of the individual DNA methylation surrogate biomarkers comprising the DNAm PhysAge with a range of clinical phenotypes in NICOLA.**

Associations are adjusted for age, sex, and white blood cell counts. Effect sizes are displayed as beta values for walking speed, grip strength, and frailty; incident rate ratios for cognitive errors, ADLs, IADLs, and Self-Rated Health; and Hazard Ratios for Mortality. Scales for DNAm surrogates of HDL, DHEAS, and peak flow are reversed so that all surrogates are expected to show the same direction of effect with the phenotypes. All associations are adjusted for age, sex, and white blood cell counts. Associations with walking speed and grip strength were also adjusted for height.

## **Figure S5a. Comparison of the prediction for aging phenotypes from the physiological health risk score (PhysRS) and Epigenetic Clocks in HRS.**

Shown in each panel is an aging phenotype and area under the receiver operating curve (AUC) from each predictor. True positive predictive percentage is shown on the y-axis and false positive percentage shown on the x-axis.

## **Figure S5b. Comparison of the prediction for aging phenotypes from the physiological health risk score (PhysRS) and Epigenetic Clocks in TILDA.**

## **Figure S5c. Comparison of the prediction for aging phenotypes from the physiological health risk score (PhysRS) and Epigenetic Clocks in NICOLA.**

**
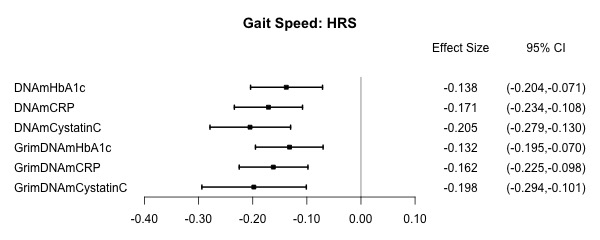

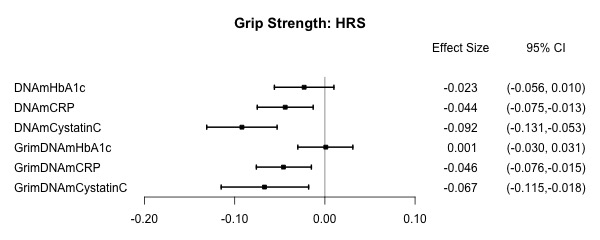
**

**
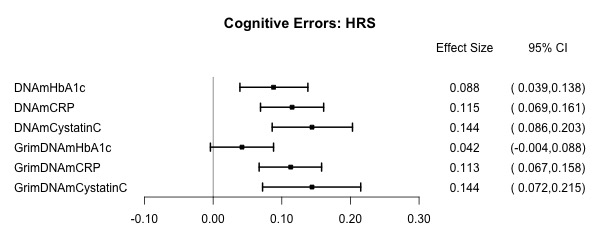

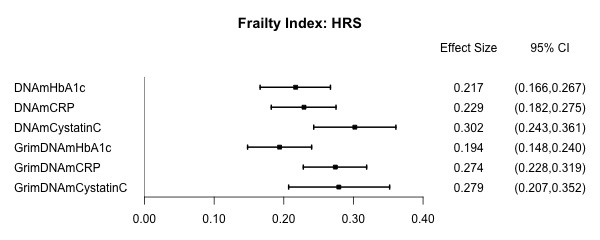
**

**
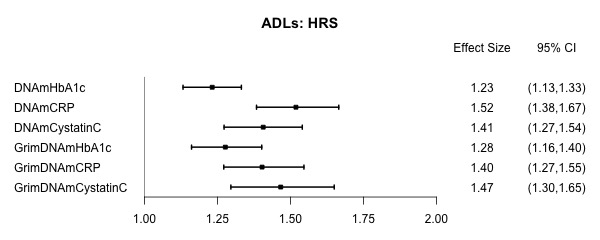

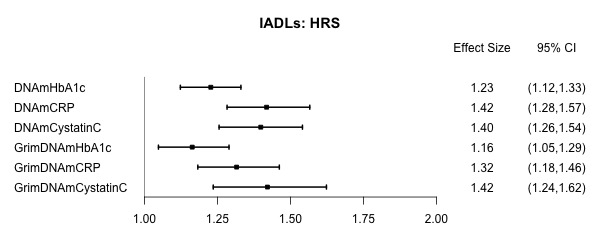
**

**
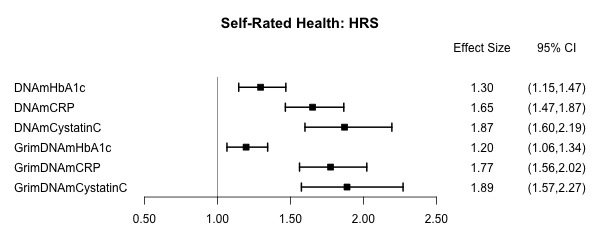

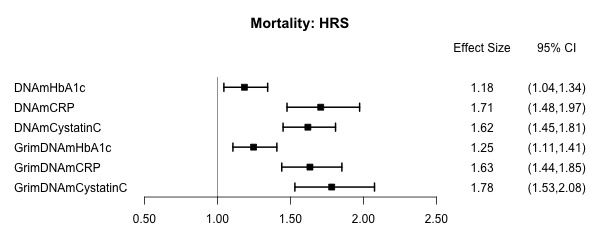
**

## **Figure S6. Comparisons of the DNAm surrogates in common between PhysAge and GrimAge in the prediction of aging phenotypes in the HRS test sample (n=1588).**

Associations are adjusted for age, sex, and race/ethnicity. Models for gait speed and grip strength were also adjusted for height.


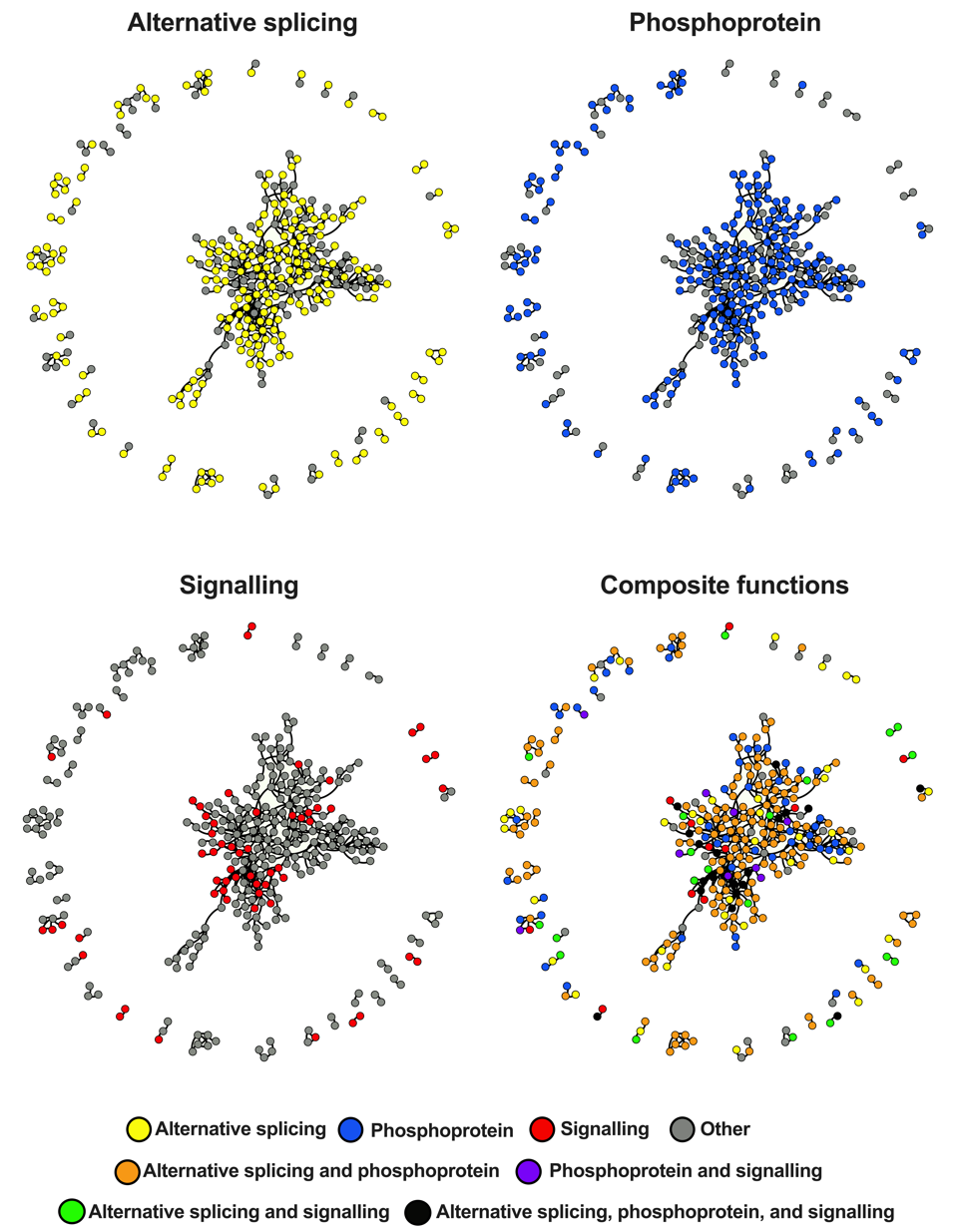


## **Figure S7. Gene network enrichment analysis of the DNAm surrogate sites comprising the DNA methylation Physiological health age (PhysAge) score.**

Gene enrichment analysis was undertaken with coding gene sets mapped from CpG sites comprising the DNAm surrogate biomarkers using StringDB *v*.12.0 and its associated databases. Each gene (node) in this network was derived by its proximity to an enriched CpG site. Genes without any interactions detected from CpG enrichment are hidden. The upper two figures display genes annotated only if they have alternative splicing transcripts (i.e. codes for more than one protein) or are a phosphoprotein (i.e. regulated at the protein-level in addition to the DNA level). The lower left subgraph highlights proteins involved in signalling (i.e. cell-cell communication). There is considerable overlap in traits, so this graphical separation serves as a visual aid. The final subgraph (lower right) illustrates the overlap between traits. Node size is uniform, edges (links between nodes) are unweighted to only display the presence of an interaction, and positioning of gene clusters is arbitrary.

## **Figure S8. Jaccard indices between gene sets indicate the overall shared content observed between gene sets, expressed as a proportion.**

The maximal Jaccard distance between two sets was approximately *J* = 0.044 suggesting that the most related sets only had an overlap of approximately 4.4%.

##
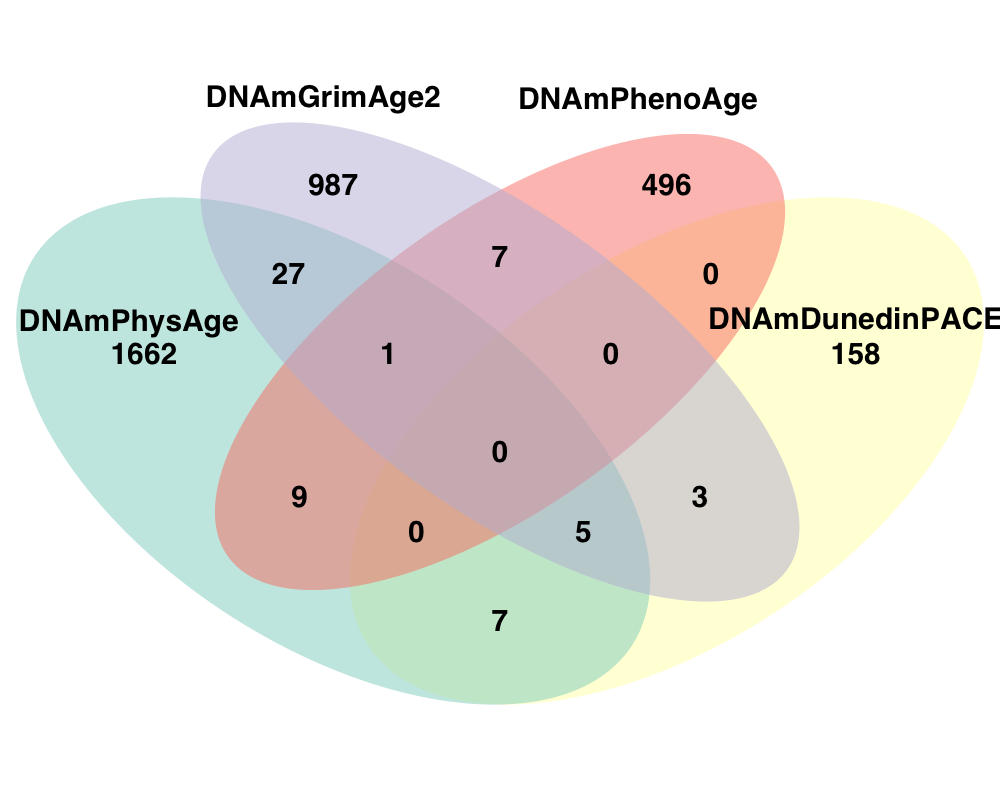


## **Figure S9. Venn Diagram describes the number of overlapping CpG sites in the 2^nd^ generation clocks.**


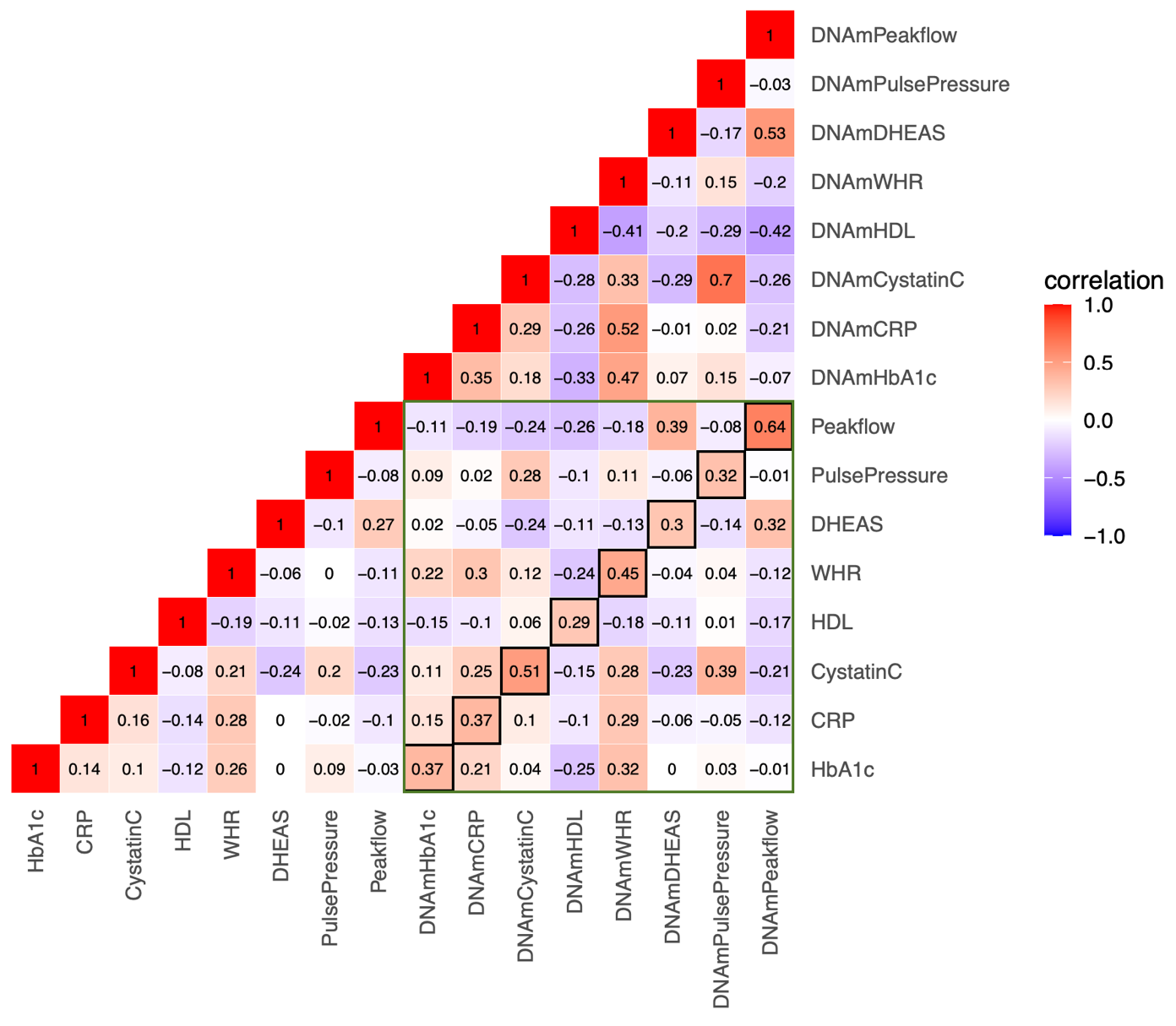


## **Figure S10. Heatmap of correlations between DNAm surrogates and their clinical biomarker in the HRS test set (n=1588).**

The green square displays correlations between the DNAm surrogate and clinical biomarkers. The diagonal of black squares shows the correlations between the specific DNAm surrogate and its respective clinical biomarker, with correlations that range from r=0.30 to r=.64. For surrogates HbA1c, CRP, Cystatin C, HDL, WHR, Peakflow, closer ties are observed for each surrogate to its target biomarker vs other biomarkers. The two exceptions are the surrogate for DHEAS, which is correlated with its clinical measure (r=.30) and also with Peakflow (r=.39), and the surrogate for Pulse Pressure, which is correlated with its clinical measure (r=.32) and also with Cystatin C (r=.39). This indicates the surrogates are exhibiting prominent specificity while DHEAS and pulse pressure are partially reflecting similar underlying processes of aging.

**
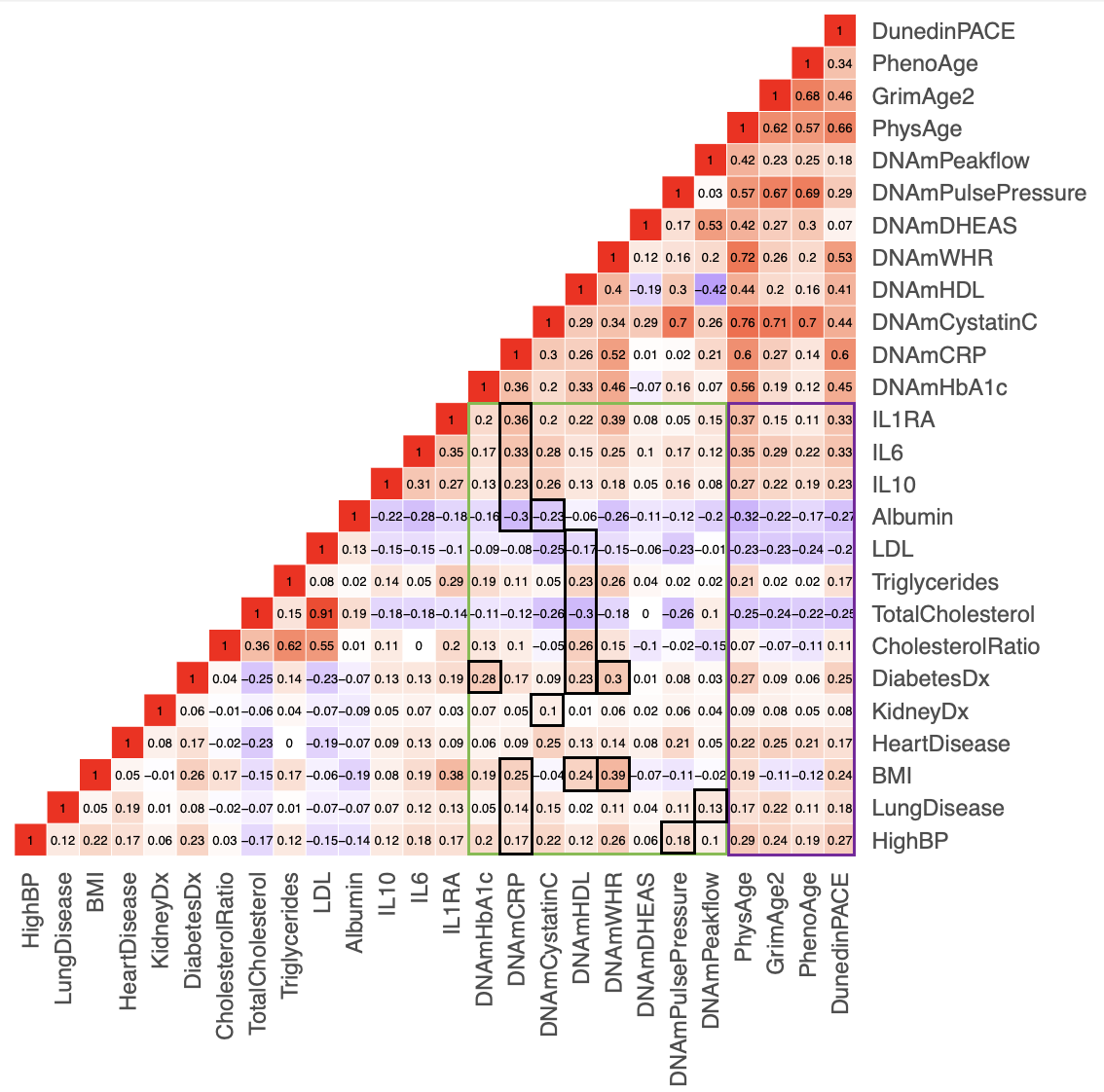
**

## **Figure S11. Heatmap of correlations between DNAm surrogates and health assessments in the HRS test set (n=1588).**

The green square shows the correlations between the DNAm surrogates and health parameters. The purple square shows the correlations between DNAm age clocks and health parameters. The health parameters include blood-based measures of inflammation (Interleukin-1 receptor antagonist (IL1RA) also indicating metabolic syndrome, Interleukin-6 (IL6), Interleukin-10 (IL10)), kidney health (Albumin), heart health (Low density lipoprotein (LDL), Triglycerides, Total Cholesterol, Cholesterol Ratio (calculated as total cholesterol divided by HDL)), a measure of body mass index (BMI in kg/m2), and self-reported diagnoses for type 2 diabetes, kidney disease, heart disease, lung disease, and hypertension/high blood pressure. Surrogates for HDL, DHEAS and peakflow are reverse coded. Measures for interleukins and triglycerides were log-transformed for normality. The heatmap shows a strong pattern of correlations between the surrogates and the specific health parameters they are intended to predict, as indicated by black squares. DNAm HbA1c and ever having been diagnosed with type 2 diabetes, DNAm WHR and high BMI, DNAm CRP and inflammatory markers IL1RA and IL6, or DNAm HDL and total cholesterol.

# **SUPPLEMENTARY TABLES**

## **Table S1a.** **Summary statistics for the biological ageing measures and health outcomes in the TILDA validation sample (n=488)**

|  | **Mean or %** | **SD** | **Min** | **Max** | **Description** |
| --- | --- | --- | --- | --- | --- |
| **Physiological Ageing Measures** | | | | | |
| Age | 62.2 | 8.35 | 50.0 | 87.0 | Chronological age |
| Female sex | 49.6% | - | - | - | Sex |
| PhysAge surrogate | 0.00 | 4.30 | -10.61 | 15.41 | DNAm surrogate sum of the standardized 8-item BRS |
| PhysAge | 68.2 | 9.63 | 42.81 | 100.40 | DNAm PhysAge |
| PhenoAge | 53.3 | 8.73 | 29.4 | 82.4 | DNAm PhenoAge |
| GrimAge2 | 68.9 | 8.47 | 46.9 | 93.5 | DNAm GrimAge2 |
| DunedinPACE | 1.05 | 0.13 | 0.78 | 1.44 | DNAm DunedinPACE |
| zPhysAge_AA | 0.00 | 1.0 | -3.04 | 3.70 | Standardized age-residualized version of the DNAm surrogate |
| zPhenoAge_AA | 0.00 | 1.0 | -2.38 | 3.00 | Standardized age-residualized version of PhenoAge |
| zGrimAge2_AA | 0.00 | 1.0 | -2.61 | 2.94 | Standardized age-residualized version of GrimAge2 |
| zDunedinPACE_AA | 0.00 | 1.0 | -2.49 | 3.20 | Standardized age-residualized version of DunedinPACE |
| **Health outcome measures** | | | | | |
| Walking speed (cms/sec) | 136.20 | 21.54 | 64.2 | 187.4 | Average of 2 timed walks at normal walking speed over 4.88 metres. |
| Mean grip strength (kgs) | 28.15 | 9.75 | 4.50 | 58.0 | Highest recorded grip strength of 4 readings |
| ADLs | 0.12 | 0.10 | 0 | 5 | ADLs included difficulties with (1) dressing, (2) walking across a room, (3) bathing or showering, (4) eating, such as cutting up food, (5) getting in or out of bed. |
| IADLs | 0.00 | 0.69 | 0 | 5 | IADLs included (1) difficulties in preparing a hot meal, (2) shopping for groceries, (3) making telephone calls, (4) taking medications, and (5) managing money. |
| Frailty index | 4.76 | 4.12 | 0 | 21 | 40-item self-report frailty index |
| MOCA errors | 11.92 | 4.93 | 0 | 17 | MOCA cognitive score, as the number correct subtracted from the total possible score of 30 |
| Self-rated health = fair/poor | 27.1% | - | - | - | Excellent/V good/Good = 0; fair/poor = 1 |
| Mortality | 10.7% | - | - | - | Hazard of all-cause mortality from 2009 to 2023 |

## **Table S1b.** **Summary statistics for the biological ageing measures and health outcomes in the NICOLA validation sample (n=1,830)**

|  | **Mean or %** | **SD** | **Min** | **Max** | **Description** |
| --- | --- | --- | --- | --- | --- |
| **Physiological Ageing Measures** | | | | | |
| Age | 64.50 | 9.09 | 50.00 | 93.4* | Chronological age |
| Female sex | 51.1% | - | - | - | Sex |
| PhysAge surrogate | 0.00 | 4.89 | -14.13 | 20.61 | DNAm surrogate sum of the standardized 8-item BRS |
| PhysAge | 68.15 | 9.63 | 40.30 | 108.77 | DNAm PhysAge |
| PhenoAge | 56.65 | 11.70 | 26.89 | 115.51 | DNAm PhenoAge |
| GrimAge2 | 68.05 | 7.95 | 49.17 | 98.35 | DNAm GrimAge2 |
| DunedinPACE | 1.00 | 0.13 | 0.63 | 1.61 | DNAm DunedinPACE |
| zPhysAge_AA | 0.00 | 1.00 | -2.48 | 4.90 | Standardized age-residualized version of the DNAm surrogate |
| zPhenoAge_AA | 0.00 | 1.00 | -2.73 | 5.48 | Standardized age-residualized version of PhenoAge |
| zGrimAge2_AA | 0.00 | 1.00 | -2.57 | 4.85 | Standardized age-residualized version of GrimAge2 |
| zDunedinPACE_AA | 0.00 | 1.00 | -2.51 | 4.37 | Standardized age-residualized version of DunedinPACE |
| **Health outcome measures** | | | | | |
| Timed Up and Go (cm/s) | 9.99 | 2.74 | 5.20 | 9.91 | Average of 2 timed walks at normal walking speed over 4.88 metres. |
| Max grip strength (kg/in^2^) | 33.45 | 11.88 | 6.40 | 9.00 | Highest recorded grip strength of 4 readings |
| ADLs | 0.21 | 0.68 | 0 | 5 | ADLs included difficulties with (1) dressing, (2) walking across a room, (3) bathing or showering, (4) eating, such as cutting up food, (5) getting in or out of bed. |
| IADLs | 0.14 | 0.53 | 0 | 5 | IADLs included (1) difficulties in preparing a hot meal, (2) shopping for groceries, (3) making telephone calls, (4) taking medications, and (5) managing money. |
| Frailty index | 7.41 | 3.15 | 2.00ᵀ | 20.2ᵀ | 40-item self-report frailty index |
| MOCA errors | 4.35 | 3.07 | 0.00 | 16.80 | MOCA cognitive score, as the number correct subtracted from the total possible score of 30 |
| Self-rated health = fair/poor | 25.6% | - | - | - | Excellent/V good/Good = 0; fair/poor = 1 |
| Mortality | 8.8% | - | - | - | Hazard of all-cause mortality from Health Assessment to 2023. |

*For NICOLA: Unable to disclose exact max age in cohort as cell count <5 but mean age of 5 oldest participants reported instead.

ᵀFor NICOLA: Unable to disclose exact min and max frailty index scores as cell count <5 but mean of 5 lowest and highest values reported instead.

## **Table S2a. Summary Measures and Area Under the Curve (AUC) for HRS**

| **Phenotype** | **Clock** | **IRR** | **LB** | **UB** | **AUC** | **diff AUC** |
| --- | --- | --- | --- | --- | --- | --- |
| ADLs | PhysRS | 1.88 | 1.73 | 2.04 | 74.26% |  |
| ADLs | PhysAge | 1.53 | 1.40 | 1.67 | 70.12% | **REF** |
| ADLs | PhenoAge | 1.21 | 1.11 | 1.32 | 66.49% | -3.63% |
| ADLs | GrimAge2 | 1.27 | 1.16 | 1.39 | 66.90% | -3.22% |
| ADLs | DunedinPACE | 1.48 | 1.36 | 1.62 | 69.89% | -0.23% |
|  |  |  |  |  |  |  |
| IADLs | PhysRS | 1.76 | 1.61 | 1.93 | 71.98% |  |
| IADLs | PhysAge | 1.45 | 1.32 | 1.60 | 68.41% | **REF** |
| IADLs | PhenoAge | 1.07 | 0.97 | 1.18 | 64.42% | -3.99% |
| IADLs | GrimAge2 | 1.26 | 1.14 | 1.40 | 66.55% | -1.86% |
| IADLs | DunedinPACE | 1.45 | 1.32 | 1.59 | 68.23% | -0.18% |
|  |  |  |  |  |  |  |
| Fair/poor Health | PhysRS | 2.07 | 1.81 | 2.38 | 70.75% |  |
| Fair/poor Health | PhysAge | 1.65 | 1.46 | 1.87 | 67.82% | **REF** |
| Fair/poor Health | PhenoAge | 1.31 | 1.17 | 1.47 | 62.83% | -4.99% |
| Fair/poor Health | GrimAge2 | 1.66 | 1.47 | 1.88 | 66.33% | -1.49% |
| Fair/poor Health | DunedinPACE | 1.53 | 1.36 | 1.72 | 66.33% | -1.49% |
|  |  |  |  |  |  |  |
|  |  | **HR** | **LB** | **UB** | **AUC** |  |
| Mortality | PhysRS | 2.08 | 1.88 | 2.29 | 82.20% |  |
| Mortality | PhysAge | 1.59 | 1.43 | 1.76 | 81.69% | **REF** |
| Mortality | PhenoAge | 1.26 | 1.14 | 1.39 | 80.12% | -1.57% |
| Mortality | GrimAge2 | 1.59 | 1.43 | 1.76 | 82.60% | 0.91% |
| Mortality | DunedinPACE | 1.49 | 1.34 | 1.66 | 81.18% | -0.51% |
|  |  |  |  |  |  |  |
|  |  | **B** | **LB** | **UB** | **AUC** |  |
| Frailty | PhysRS | 0.34 | 0.31 | 0.38 | 75.40% |  |
| Frailty | PhysAge | 0.23 | 0.20 | 0.27 | 72.47% | **REF** |
| Frailty | PhenoAge | 0.09 | 0.06 | 0.13 | 67.72% | -4.75% |
| Frailty | GrimAge2 | 0.18 | 0.15 | 0.22 | 70.25% | -2.22% |
| Frailty | DunedinPACE | 0.22 | 0.18 | 0.25 | 70.38% | -2.09% |
|  |  |  |  |  |  |  |
| Grip strength | PhysRS | -0.80 | -1.15 | -0.44 | 89.74% |  |
| Grip strength | PhysAge | -0.54 | -0.88 | -0.20 | 89.50% | **REF** |
| Grip strength | PhenoAge | -0.49 | -0.81 | -0.17 | 89.60% | 0.10% |
| Grip strength | GrimAge2 | -0.58 | -0.91 | -0.25 | 89.51% | 0.01% |
| Grip strength | DunedinPACE | -0.26 | -0.60 | 0.08 | 89.51% | 0.01% |
|  |  |  |  |  |  |  |
| Walking speed | PhysRS | -8.54 | -10.12 | -6.97 | 75.40% |  |
| Walking speed | PhysAge | -5.13 | -6.57 | -3.69 | 72.03% | **REF** |
| Walking speed | PhenoAge | -1.23 | -2.67 | 0.20 | 69.40% | -2.63% |
| Walking speed | GrimAge2 | -3.42 | -4.94 | -1.90 | 70.79% | -1.24% |
| Walking speed | DunedinPACE | -4.30 | -5.67 | -2.93 | 71.62% | -0.41% |
|  |  |  |  |  |  |  |
| CogErrors | PhysRS | 0.80 | 0.60 | 1.01 | 73.19% |  |
| CogErrors | PhysAge | 0.57 | 0.37 | 0.77 | 71.53% | **REF** |
| CogErrors | PhenoAge | 0.12 | -0.08 | 0.31 | 70.62% | -0.91% |
| CogErrors | GrimAge2 | 0.57 | 0.37 | 0.76 | 71.79% | 0.26% |
| CogErrors | DunedinPACE | 0.66 | 0.46 | 0.85 | 71.14% | -0.39% |

Effect sizes are displayed as incident rate ratios for ADLs, IADLs, and Self-Rated Health; Hazard Ratios for Mortality; and beta values for walking speed, grip strength, cognitive errors, and frailty. IRR = Incident Rate Ratios; LB = Lower Bound of 95% Confidence Interval; UB = Upper Bound of 95% Confidence Interval; AUC = Area under the receiver operating curve; diff AUC = difference in AUC; REF = reference for AUC difference comparison; HR = Hazard Ratio; B = beta coefficient.

For the calculation of AUCs, first continuous variables were dichotomized using median values, then logistic regression analyses were used to estimate the area under the receiver operating curve.

## **Table S2b. Summary Measures and Area Under the Curve (AUC) for TILDA**

| **Phenotype** | **Clock** | **IRR** | **LB** | **UB** | **AUC** | **diff AUC** |
| --- | --- | --- | --- | --- | --- | --- |
| ADLs | PhysRS | N/A | N/A | N/A | N/A |  |
| ADLs | PhysAge | 1.56 | 1.22 | 1.98 | 71.50% | **REF** |
| ADLs | PhenoAge | 1.19 | 0.92 | 1.53 | 67.06% | -4.40% |
| ADLs | GrimAge2 | 1.52 | 1.17 | 1.99 | 69.46% | -2.04% |
| ADLs | DunedinPACE | 1.56 | 1.23 | 1.98 | 71.42% | -0.08% |
|  |  |  |  |  |  |  |
| IADLs | PhysRS | N/A | N/A | N/A | N/A |  |
| IADLs | PhysAge | 1.66 | 1.26 | 2.17 | 76.16% | **REF** |
| IADLs | PhenoAge | 1.15 | 0.86 | 1.53 | 69.24% | -6.92% |
| IADLs | GrimAge2 | 1.52 | 1.11 | 2.08 | 72.76% | -3.40% |
| IADLs | DunedinPACE | 1.77 | 1.36 | 2.29 | 77.95% | 1.79% |
|  |  |  |  |  |  |  |
| Fair/poor Health | PhysRS | N/A | N/A | N/A | N/A |  |
| Fair/poor Health | PhysAge | 1.59 | 1.27 | 1.99 | 65.22% | **REF** |
| Fair/poor Health | PhenoAge | 1.60 | 1.27 | 2.01 | 64.29% | -0.93% |
| Fair/poor Health | GrimAge2 | 1.77 | 1.41 | 2.23 | 65.80% | 0.58% |
| Fair/poor Health | DunedinPACE | 1.74 | 1.39 | 2.19 | 66.56% | 1.34% |
|  |  |  |  |  |  |  |
| CogErrors | PhysRS | N/A | N/A | N/A | N/A |  |
| CogErrors | PhysAge | 1.13 | 1.06 | 1.20 | 65.90% | **REF** |
| CogErrors | PhenoAge | 1.09 | 1.03 | 1.16 | 66.46% | 0.56% |
| CogErrors | GrimAge2 | 1.15 | 1.08 | 1.22 | 67.38% | 1.48% |
| CogErrors | DunedinPACE | 1.16 | 1.10 | 1.23 | 67.24% | 1.34% |
|  |  |  |  |  |  |  |
|  |  | **HR** | **LB** | **UB** | **AUC** |  |
| Mortality | PhysRS | N/A | N/A | N/A | N/A |  |
| Mortality | PhysAge | 1.28 | 1.02 | 1.62 | 87.71% | **REF** |
| Mortality | PhenoAge | 1.06 | 0.81 | 1.38 | 86.66% | -1.05% |
| Mortality | GrimAge2 | 1.54 | 1.15 | 2.06 | 87.49% | -0.22% |
| Mortality | DunedinPACE | 1.47 | 1.16 | 1.86 | 88.07% | 0.36% |
|  |  |  |  |  |  |  |
|  |  | **B** | **LB** | **UB** | **AUC** |  |
| Frailty | PhysRS | N/A | N/A | N/A | N/A |  |
| Frailty | PhysAge | 0.26 | 0.19 | 0.33 | 74.15% | **REF** |
| Frailty | PhenoAge | 0.16 | 0.09 | 0.23 | 71.70% | -2.45% |
| Frailty | GrimAge2 | 0.19 | 0.12 | 0.26 | 73.81% | -0.34% |
| Frailty | DunedinPACE | 0.22 | 0.15 | 0.30 | 73.96% | -0.19% |
|  |  |  |  |  |  |  |
| Grip strength | PhysRS | N/A | N/A | N/A | N/A |  |
| Grip strength | PhysAge | -0.47 | -0.99 | 0.05 | 94.40% | **REF** |
| Grip strength | PhenoAge | 0.12 | -0.40 | 0.64 | 94.17% | -0.23% |
| Grip strength | GrimAge2 | -0.58 | -1.12 | -0.05 | 94.38% | -0.02% |
| Grip strength | DunedinPACE | -0.50 | -1.02 | 0.02 | 94.31% | -0.09% |
|  |  |  |  |  |  |  |
| Walking speed | PhysRS | N/A | N/A | N/A | N/A |  |
| Walking speed | PhysAge | -4.46 | -6.27 | -2.66 | 72.28% | **REF** |
| Walking speed | PhenoAge | -2.45 | -4.32 | -0.58 | 72.04% | -0.24% |
| Walking speed | GrimAge2 | -5.07 | -6.94 | -3.20 | 73.48% | 1.20% |
| Walking speed | DunedinPACE | -4.53 | -6.39 | -2.68 | 72.31% | -0.19% |

Effect sizes are displayed as incident rate ratios for ADLs, IADLs, and Self-Rated Health; Hazard Ratios for Mortality; and beta values for walking speed, grip strength, cognitive errors, and frailty. IRR = Incident Rate Ratios; LB = Lower Bound of 95% Confidence Interval; UB = Upper Bound of 95% Confidence Interval; AUC = Area under the receiver operating curve; diff AUC = difference in AUC; REF = reference for AUC difference comparison; HR = Hazard Ratio; B = beta coefficient.

For the calculation of AUCs, first continuous variables were dichotomized using median values, then logistic regression analyses were used to estimate the area under the receiver operating curve.

## **Table S2c. Summary Measures and Area Under the Curve (AUC) for NICOLA**

| **Phenotype** | **Clock** | **IRR** | **LB** | **UB** | **AUC** | **diff AUC** |
| --- | --- | --- | --- | --- | --- | --- |
| ADLs | PhysRS | N/A | N/A | N/A | N/A | N/A |
| ADLs | PhysAge | 1.84 | 1.61 | 2.11 | 66.11% | **REF** |
| ADLs | PhenoAge | 1.54 | 1.32 | 1.79 | 61.82% | -4.29% |
| ADLs | GrimAge2 | 2.00 | 1.69 | 2.36 | 63.51% | -2.60% |
| ADLs | DunedinPACE | 1.72 | 1.54 | 1.90 | 65.25% | -0.86% |
|  |  |  |  |  |  |  |
| IADLs | PhysRS | N/A | N/A | N/A | N/A | N/A |
| IADLs | PhysAge | 1.64 | 1.38 | 1.94 | 66.97% | **REF** |
| IADLs | PhenoAge | 1.22 | 1.00 | 1.48 | 63.02% | -3.95% |
| IADLs | GrimAge2 | 1.91 | 1.54 | 2.35 | 66.62% | -0.35% |
| IADLs | DunedinPACE | 1.56 | 1.37 | 1.78 | 67.04% | 0.07% |
|  |  |  |  |  |  |  |
| Fair/poor Health | PhysRS | N/A | N/A | N/A | N/A | N/A |
| Fair/poor Health | PhysAge | 1.94 | 1.65 | 2.27 | 63.81% | **REF** |
| Fair/poor Health | PhenoAge | 1.42 | 1.19 | 1.69 | 59.66% | -4.15% |
| Fair/poor Health | GrimAge2 | 2.40 | 1.96 | 2.94 | 64.11% | 0.30% |
| Fair/poor Health | DunedinPACE | 1.76 | 1.55 | 1.99 | 64.18% | 0.37% |
|  |  |  |  |  |  |  |
| CogErrors | PhysRS | N/A | N/A | N/A | N/A | N/A |
| CogErrors | PhysAge | 1.11 | 1.06 | 1.16 | 66.74% | **REF** |
| CogErrors | PhenoAge | 1.06 | 1.00 | 1.11 | 66.25% | -0.49% |
| CogErrors | GrimAge2 | 1.11 | 1.04 | 1.17 | 66.38% | -0.36% |
| CogErrors | DunedinPACE | 1.10 | 1.06 | 1.14 | 67.07% | 0.33% |
|  |  |  |  |  |  |  |
|  |  | **HR** | **LB** | **UB** | **AUC** |  |
| Mortality | PhysRS | N/A | N/A | N/A | N/A | N/A |
| Mortality | PhysAge | 1.55 | 1.24 | 1.93 | 81.36% | **REF** |
| Mortality | PhenoAge | 1.32 | 1.03 | 1.70 | 81.35% | -0.01% |
| Mortality | GrimAge2 | 2.27 | 1.74 | 2.97 | 83.21% | 1.85% |
| Mortality | DunedinPACE | 1.45 | 1.22 | 1.72 | 81.83% | 0.47% |
|  |  |  |  |  |  |  |
|  |  | **B** | **LB** | **UB** | **AUC** |  |
| Frailty | PhysRS | N/A | N/A | N/A | N/A | N/A |
| Frailty | PhysAge | 0.15 | 0.12 | 0.17 | 68.70% | **REF** |
| Frailty | PhenoAge | 0.08 | 0.06 | 0.11 | 65.13% | -3.57% |
| Frailty | GrimAge2 | 0.17 | 0.14 | 0.21 | 68.55% | -0.15% |
| Frailty | DunedinPACE | 0.12 | 0.11 | 0.14 | 69.89% | 1.19% |
|  |  |  |  |  |  |  |
| Grip strength | PhysRS | N/A | N/A | N/A | N/A | N/A |
| Grip strength | PhysAge | -0.60 | -1.10 | -0.09 | 92.47% | **REF** |
| Grip strength | PhenoAge | -0.16 | -0.75 | 0.44 | 92.45% | -0.02% |
| Grip strength | GrimAge2 | -1.37 | -2.02 | -0.72 | 92.67% | 0.20% |
| Grip strength | DunedinPACE | -0.41 | -0.80 | -0.02 | 92.46% | -0.01% |
|  |  |  |  |  |  |  |
| TUG | PhysRS | N/A | N/A | N/A | N/A | N/A |
| TUG | PhysAge | 0.68 | 0.50 | 0.87 | 69.88% | **REF** |
| TUG | PhenoAge | 0.44 | 0.21 | 0.66 | 69.27% | -0.61% |
| TUG | GrimAge2 | 0.83 | 0.59 | 1.07 | 70.38% | 0.50% |
| TUG | DunedinPACE | 0.48 | 0.34 | 0.62 | 70.03% | 0.15% |

Effect sizes are displayed as incident rate ratios for ADLs, IADLs, and Self-Rated Health; Hazard Ratios for Mortality; and beta values for timed-up-and-go (TUG), grip strength, cognitive errors, and frailty. IRR = Incident Rate Ratios; LB = Lower Bound of 95% Confidence Interval; UB = Upper Bound of 95% Confidence Interval; AUC = Area under the receiver operating curve; diff AUC = difference in AUC; REF = reference for AUC difference comparison; HR = Hazard Ratio; B = beta coefficient.

For the calculation of AUCs, first continuous variables were dichotomized using median values, then logistic regression analyses were used to estimate the area under the receiver operating curve.

## **Table S3. Comparisons of DNAm surrogates used to develop both PhysAge and GrimAge2**

|  | PhysAge | GrimAge2 | Overlapping | Correlation between PhysAge and GrimAge2 versions |
| --- | --- | --- | --- | --- |
|  | # CpGs | # CpGs | # CpGs |  |
| DNAmCRP | 185 | 132 | 3 | 0.57 |
| DNAmCystatinC | 238 | 87 | 2 | 0.70 |
| DNAmHbA1c | 233 | 86 | 4 | 0.55 |

## **Table S4. String DB links for coding gene sets that mapped back to CpG surrogate sets**

##

## **Table S5. Simulations of DNAm surrogates with PhysAge of 60 for four men and PhysAge of 80 for four women**

|  | **Men with PhysAge ~60** | | | | **Women with PhysAge ~80** | | | |
| --- | --- | --- | --- | --- | --- | --- | --- | --- |
| **Age Measure** | **M1** | **M2** | **M3** | **M4** | **W1** | **W2** | **W3** | **W4** |
| Chronological Age | 77.4 | 70.5 | 57.1 | 52.8 | 86.6 | 93.4 | 71.5 | 75.6 |
| DNAm PhysAge | 60.6 | 59.8 | 60.6 | 60.8 | 80.0 | 80.6 | 80.8 | 80.7 |
| DNAm HbA1c | 62.4 | 59.5 | 79.9 | 66.8 | 75.5 | 62.8 | 67.3 | 83.6 |
| DNAm CRP | 53.4 | 60.2 | 60.8 | 73.4 | 67.6 | 64.5 | 88.5 | 82.9 |
| DNAm CystatinC | 72.8 | 56.6 | 52.8 | 56.6 | 80.8 | 85.4 | 75.0 | 69.6 |
| DNAm HDL | 70.0 | 74.2 | 72.3 | 80.9 | 70.1 | 58.5 | 71.2 | 61.5 |
| DNAm WHR | 53.3 | 55.6 | 67.1 | 59.2 | 75.2 | 66.8 | 81.0 | 72.1 |
| DNAm DHEAS | 51.9 | 68.5 | 60.6 | 54.0 | 73.0 | 96.9 | 62.3 | 75.9 |
| DNAm PulsePressure | 77.6 | 70.5 | 56.3 | 60.4 | 73.4 | 83.6 | 73.0 | 76.0 |
| DNAm Peakflow | 58.9 | 51.8 | 50.2 | 49.9 | 79.4 | 79.4 | 80.3 | 76.5 |

Notes: Red values indicate where the surrogate age is older than chronological age.

# **SI References**

1. M. E. Levine *et al.*, An epigenetic biomarker of aging for lifespan and healthspan. *bioRxiv* (2018).

2. A. T. Lu *et al.*, DNA methylation GrimAge strongly predicts lifespan and healthspan. *Aging (Albany NY)* **11**, 303-327 (2019).

3. D. W. Belsky *et al.*, DunedinPACE, a DNA methylation biomarker of the pace of aging. *Elife* **11** (2022).

4. F. J. Crimmins EM, Thyagarajan B, Weir D. (2017) Venous blood collection and assay protocol in the 2016 Health and Retirement Study 2016 Venous Blood Study (VBS). .

5. C. McCrory *et al.*, How does socio-economic position (SEP) get biologically embedded? A comparison of allostatic load and the epigenetic clock(s). *Psychoneuroendocrinology* **104**, 64-73 (2019).

6. C. Neville *et al.*, Cohort profile: the Northern Ireland Cohort for the Longitudinal Study of Ageing (NICOLA). *International Journal of Epidemiology*, dyad026 (2023).

7. F. Kee, Neville, C., McGuinness, B., & Hogg, R (2021) Objective Measures of Health and Wellbeing of Older Adults in Northern Ireland - The NICOLA study Wave 1.

8. L. J. Smyth *et al.*, An investigation into DNA methylation patterns associated with risk preference in older individuals. *Epigenetics* **17**, 1159-1172 (2022).

9. C. Potter *et al.*, Cohort profile: DNA methylation in the Northern Ireland Cohort for the Longitudinal Study of Ageing (NICOLA) - recruitment and participant characteristics. *BMJ Open* **14**, e085652 (2024).

10. D. Podsiadlo, S. Richardson, The timed “Up & Go”: a test of basic functional mobility for frail elderly persons. *Journal of the American geriatrics Society* **39**, 142-148 (1991).

11. K. Rockwood, A. Mitnitski, Frailty in Relation to the Accumulation of Deficits. *The Journals of Gerontology: Series A* **62**, 722-727 (2007).

12. M.-J. McKelvie *et al.*, Frailty on the island of Ireland: evidence from the NICOLA and TILDA studies. *European Journal of Public Health* 10.1093/eurpub/ckae046 (2024).

13. M. Ward *et al.*, Linking death registration and survey data: Procedures and cohort profile for The Irish Longitudinal Study on Ageing (TILDA). *HRB Open Res* **3**, 43 (2020).

14. D. Szklarczyk *et al.*, The STRING database in 2023: protein-protein association networks and functional enrichment analyses for any sequenced genome of interest. *Nucleic Acids Res* **51**, D638-d646 (2023).

15. M. Bastian, S. Heymann, M. Jacomy (2009) Gephi: an open source software for exploring and manipulating networks. in *Proceedings of the international AAAI conference on web and social media*, pp 361-362.
